# Supplementary material for: Minimally-invasive implantable device enhances brain cancer suppression
Source: EMBO Mol Med. 2024 Jun 20;16(7):1704–16. doi: 10.1038/s44321-024-00091-5 (PMC11250787; doi:10.1038/s44321-024-00091-5)
Supplement: Supplementary file 1 — Appendix [file 44321_2024_91_MOESM1_ESM.pdf]

Appendix

Appendix Table S1 1
Appendix Figure S1 2
Appendix Figure S2 3
Appendix Figure S3 4
Appendix Figure S4 5
Appendix Figure S5 6
Appendix Figure S6 7
Appendix Figure S7 8
Appendix Figure S8 9
Appendix Figure S9 10
Appendix Figure S10 11
Appendix Figure S11 12
Appendix Figure S12 13
Appendix Figure S13 14
Appendix Figure S14 15
Appendix Figure S15 16

Appendix Table S1

patient ID	WHO grade	Histology	Molecular background	Prior treatment with radiation
T-26	III	Anaplastic astrocytoma	IDH1(-); MGMT gene promoter methylation(+); PTEN, TSC2 deletion; CDK4, CDK6, EGFR, MET, SMO, MDM4, TERT amplification; FGFR3--TACC3, PIK3CAp.K111E, PTENp.I101T, BCORp.A606fs cancer driver genes mutation	These is no radiotherapy before the tumor is surgically removed.
T-36	IV	Glioblastoma	IDH1(-); TERT promoter region mutation; MGMT gene promoter methylation(+); BRCA2, CDKN2A, CDKN2B, PTEN deletion; CDK6, SMO amplification, PIK3CAp.H1047Y, STAG2p.E470 cancer driver gene mutation	These is no radiotherapy before the tumor is surgically removed.
T-51	I	Meningiomas	NF2p.A451fs cancer driver gene mutation	These is no radiotherapy before the tumor is surgically removed.
T-59	IV	Glioblastoma	IDH1(-); TERT promoter region mutation; MGMT gene promoter methylation (+); SMO amplification; TP53p.R280K, BRCA2p.N289H, CARD11p.K83M, NOTCH2p.H107P cancer driver genes mutation	These is no radiotherapy before the tumor is surgically removed.
T-91	II	Oligodendroglioma	IDH1(+); MGMT gene promoter methylation(-)	These is no radiotherapy before the tumor is surgically removed.

Appendix Figure S1. **(a)** Output voltages between Swiss-roll devices with different numbers of turns. **(b)** Temperature changes of devices with different loops after 8 hours of

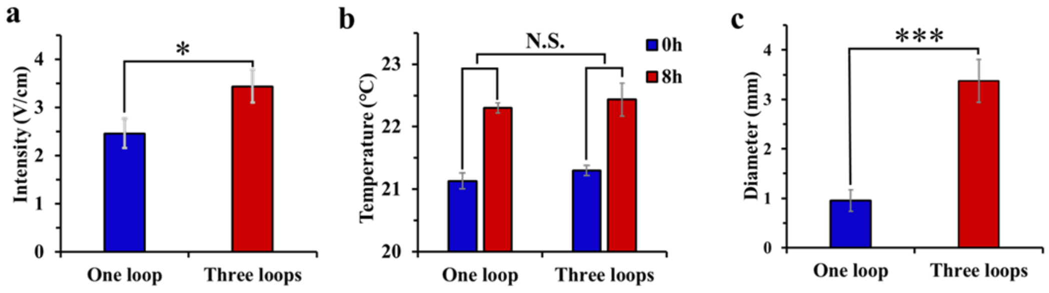

ultrasonic excitation. **(c)** The bottom diameters between Swiss-roll devices with different numbers of turns. *: p-value < 0.05; **: p-value < 0.01; ***: p-value < 0.001.

Appendix Figure S2

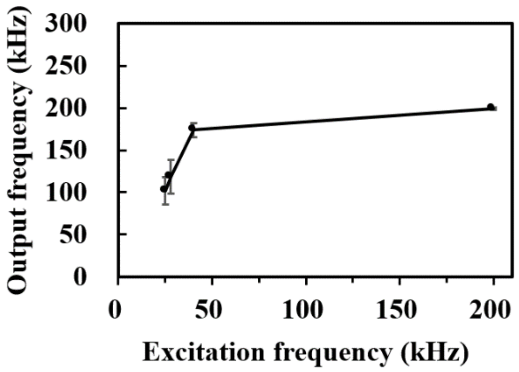

Appendix Figure S2. Tuning output frequency of the Swiss-roll device by changing the excitation ultrasound frequency.

Appendix Figure S3

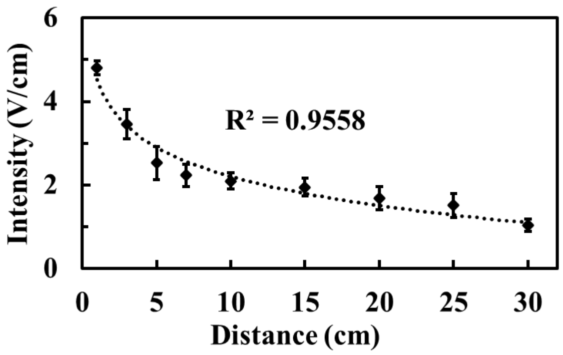

Appendix Figure S3. Fitted curve of the attenuation of output voltage with increasing transmission distance.

Appendix Figure S4

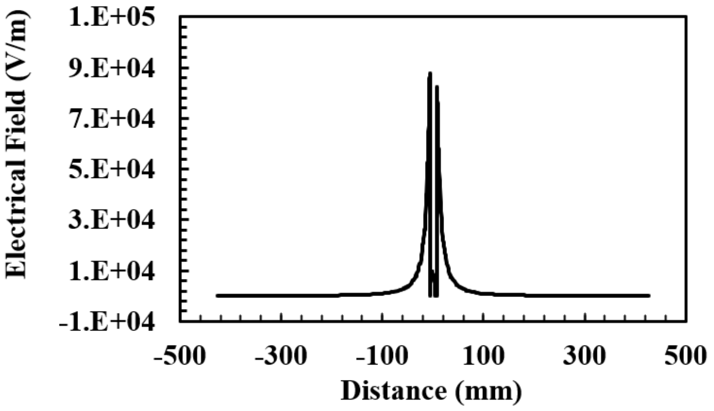

Appendix Figure S4. Simulation of horizontal axis electric field distribution of MIBTS implanted in the brain

Appendix Figure S5

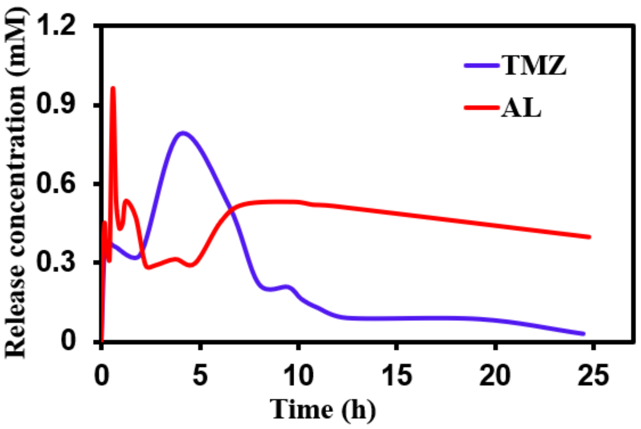

Appendix Figure S5. Drug release profiles of TMZ and AL over 24 hours.

Appendix Figure S6

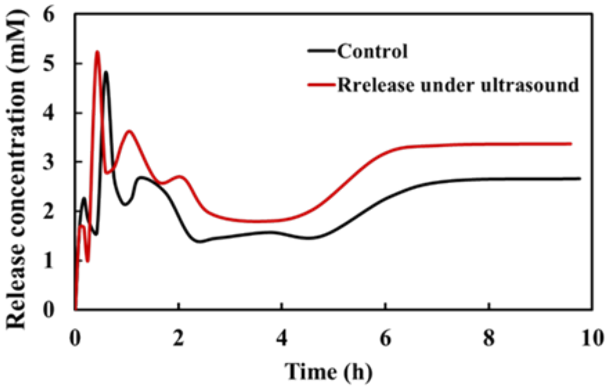

Appendix Figure S6. Drug release profile of control and AL-coated MIBTS within 10 hours.

Appendix Figure S7

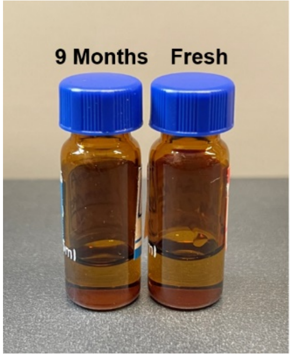

Appendix Figure S7. The ISFC formula solution are stored stable for more than 9 months without observed changes in physical appearance.

Appendix Figure S8

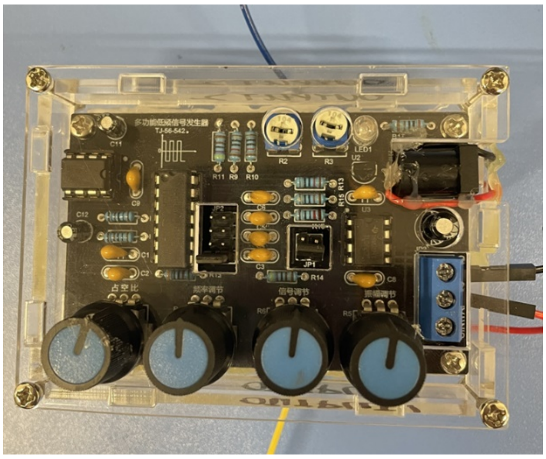

Appendix Figure S8. The homemade voltage regulation control unit. It is used to connect the patient-tailored TTF and the chemotherapy response platform to construct the gradient voltage profiles.

Appendix Figure S9

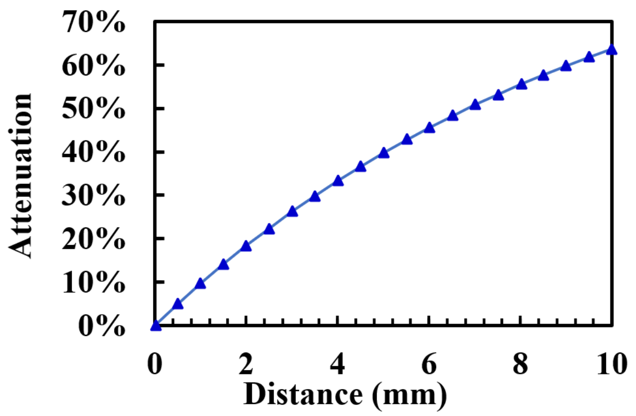

Appendix Figure S9. Ultrasound attenuation under different skull thicknesses

Appendix Figure S10

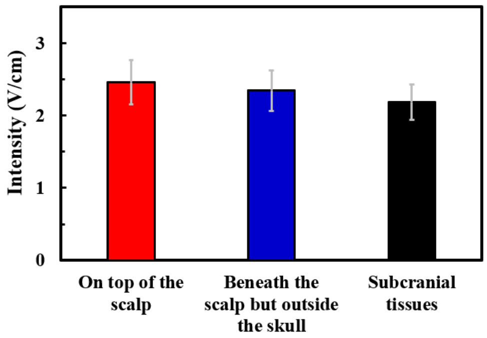

Appendix Figure S10. Device output at different implant locations

Appendix Figure S11

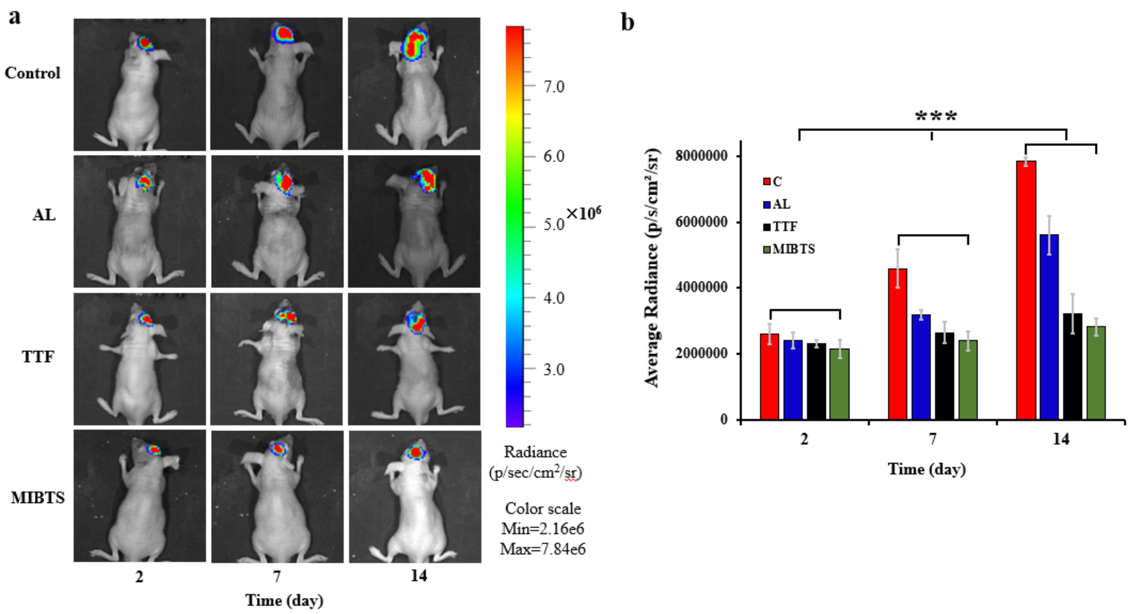

Appendix Figure S11. Detection of bioluminescence in mice. (a) In vivo fluorescence imaging of mice in each group. (b) Statistical results of average fluorescence intensity within 14 days,
***$p<0.001$.

Appendix Figure S12

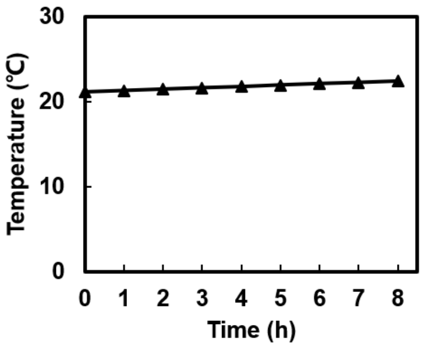

Appendix Figure S12. Mild and safe temperature rise after continuous treatment with MIBTS for several hours. The distance between the equipment and the ultrasonic source is 10mm, and the ultrasonic power is 0.4W/cm^2.

Appendix Figure S13

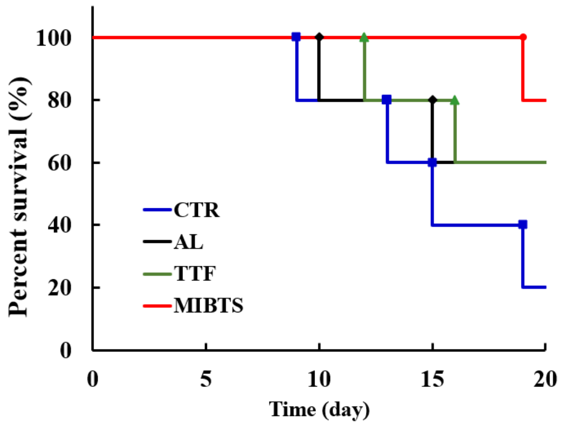

Appendix Figure S13. Survival curves of four groups of mice with different therapy strategies.

Appendix Figure S14

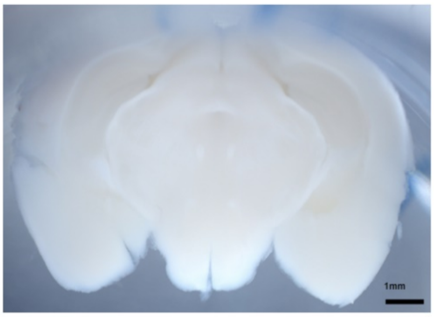

Appendix Figure S14. No obvious coagulation and necrosis in the brain tissue were found after MIBTS treatment.

Appendix Figure S15

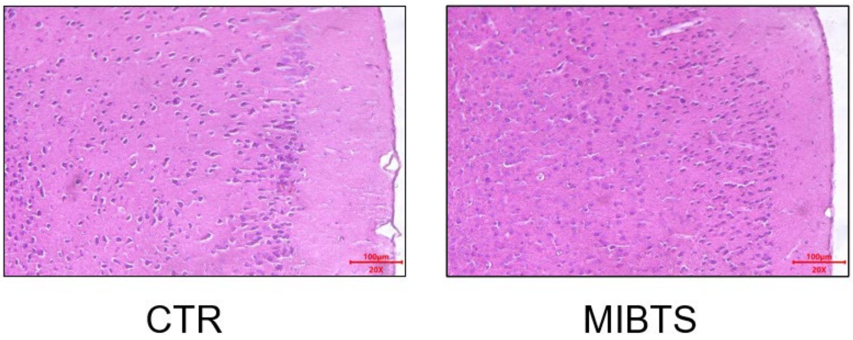

Appendix Figure S15. Long-term compatibility exploration of implanted MIBTS device after months of implantation. Inflammatory cell infiltration by hematoxylin and eosin (H&E) staining. No significant increase in leukomonocyte, macrophage, and polymorphonuclear leukocyte infiltration were observed in the MIBTS implanted animals. Scale bar 100 μm.
