# Supplementary figures and images for: Minimally-invasive implantable device enhances brain cancer suppression
Source: EMBO Mol Med. 2024 Jun 20;16(7):1704–16. doi: 10.1038/s44321-024-00091-5 (PMC11250787; doi:10.1038/s44321-024-00091-5)

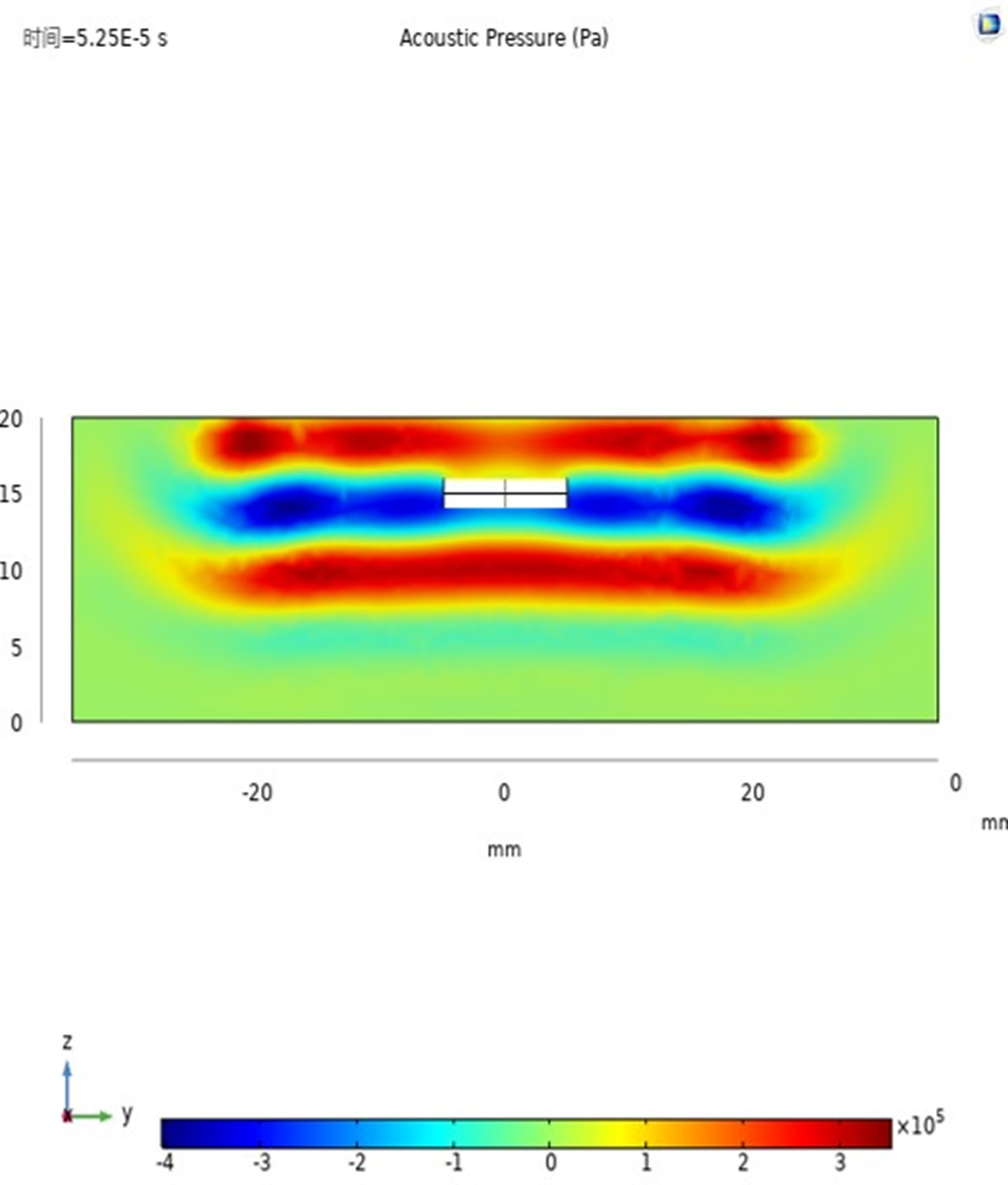

Supplement: Supplementary file 5 — Source data Fig. 3 [file 44321_2024_91_MOESM5_ESM.zip › Fig 3E.jpg]

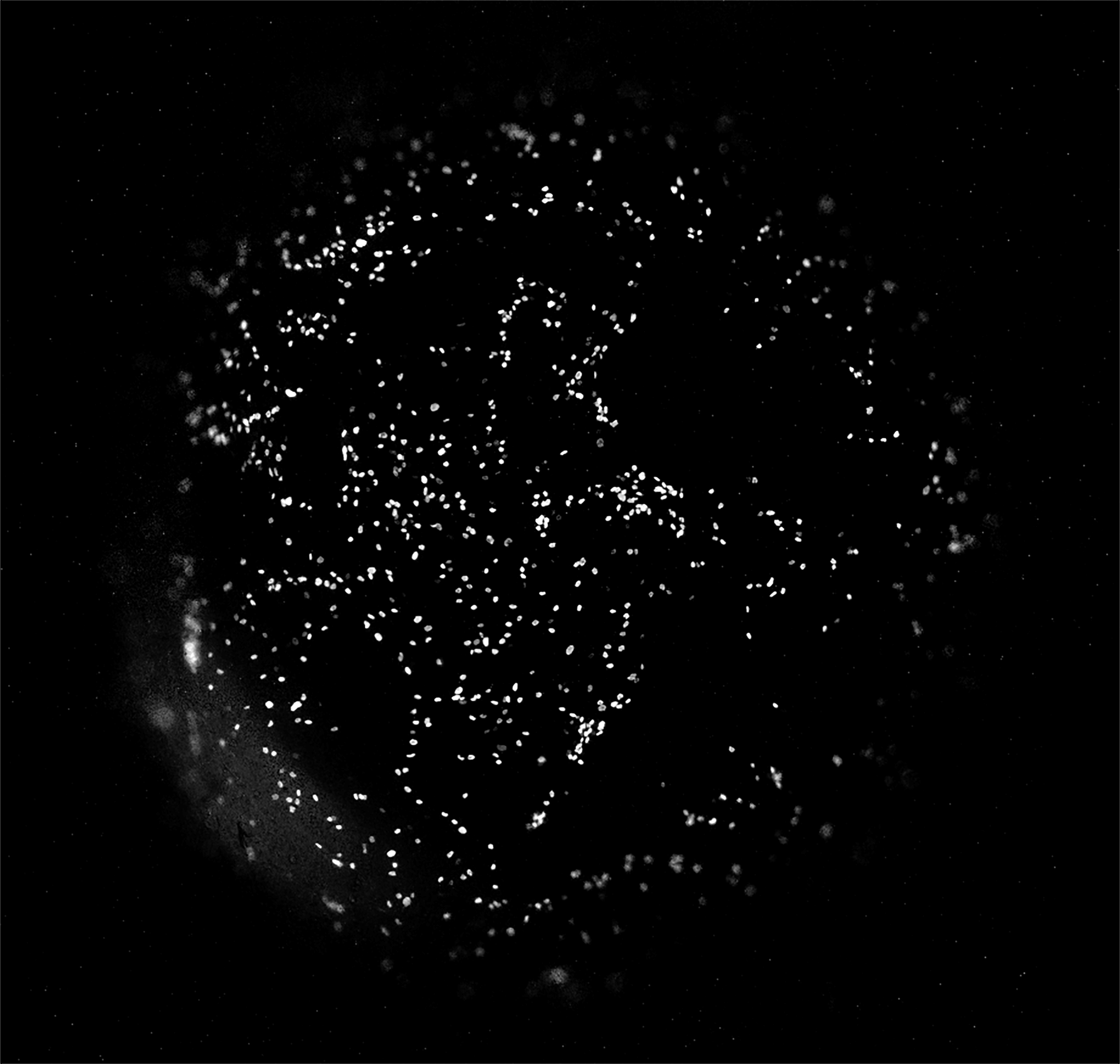

Supplement: Supplementary file 7 — Source data Fig. 5 [file 44321_2024_91_MOESM7_ESM.zip › Figure 5F/109.28V-488.tif]

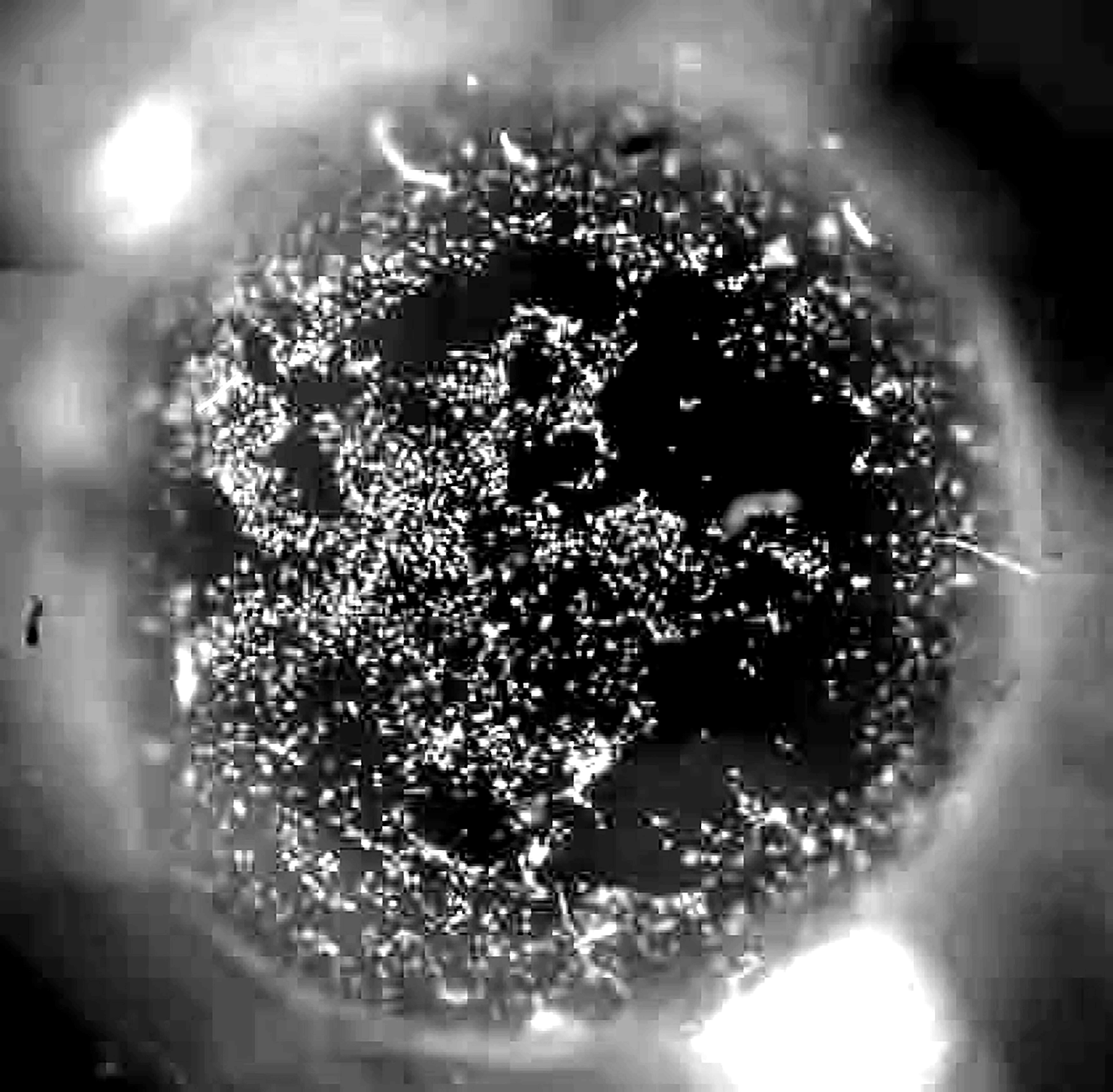

Supplement: Supplementary file 7 — Source data Fig. 5 [file 44321_2024_91_MOESM7_ESM.zip › Figure 5F/109.28V-DAPI.tif]

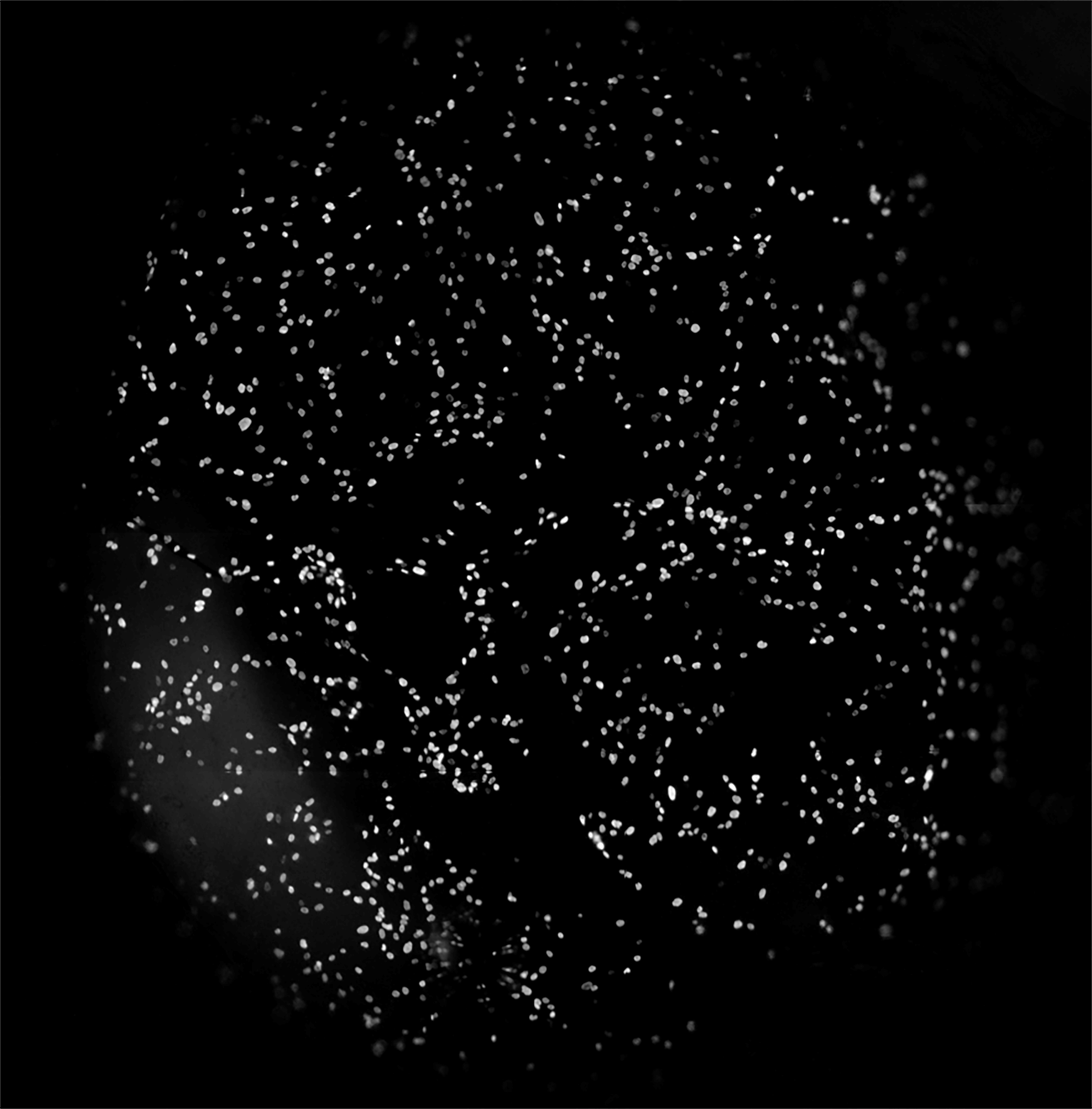

Supplement: Supplementary file 7 — Source data Fig. 5 [file 44321_2024_91_MOESM7_ESM.zip › Figure 5F/14.94V-488.tif]

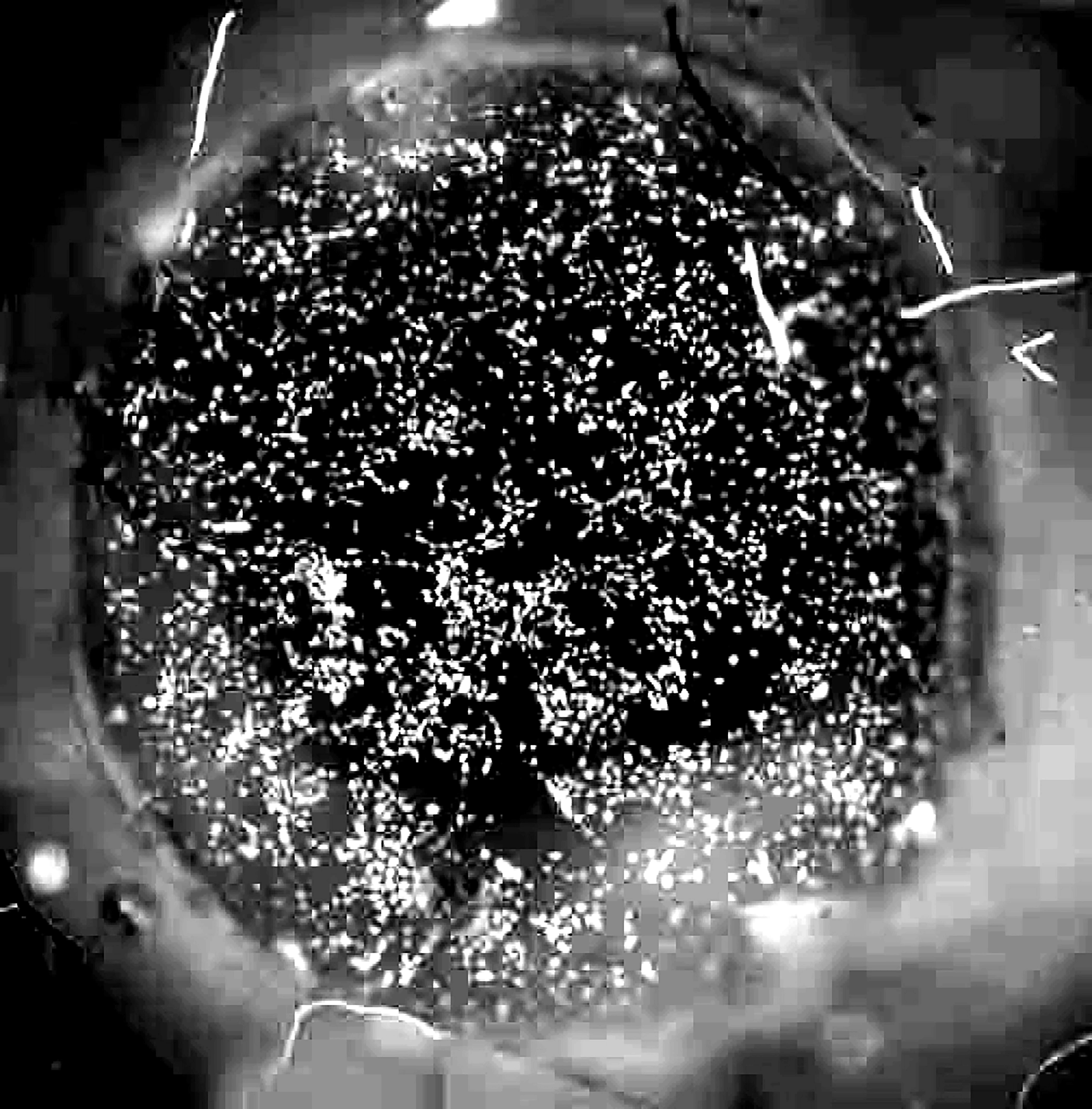

Supplement: Supplementary file 7 — Source data Fig. 5 [file 44321_2024_91_MOESM7_ESM.zip › Figure 5F/14.94V-DAPI.tif]

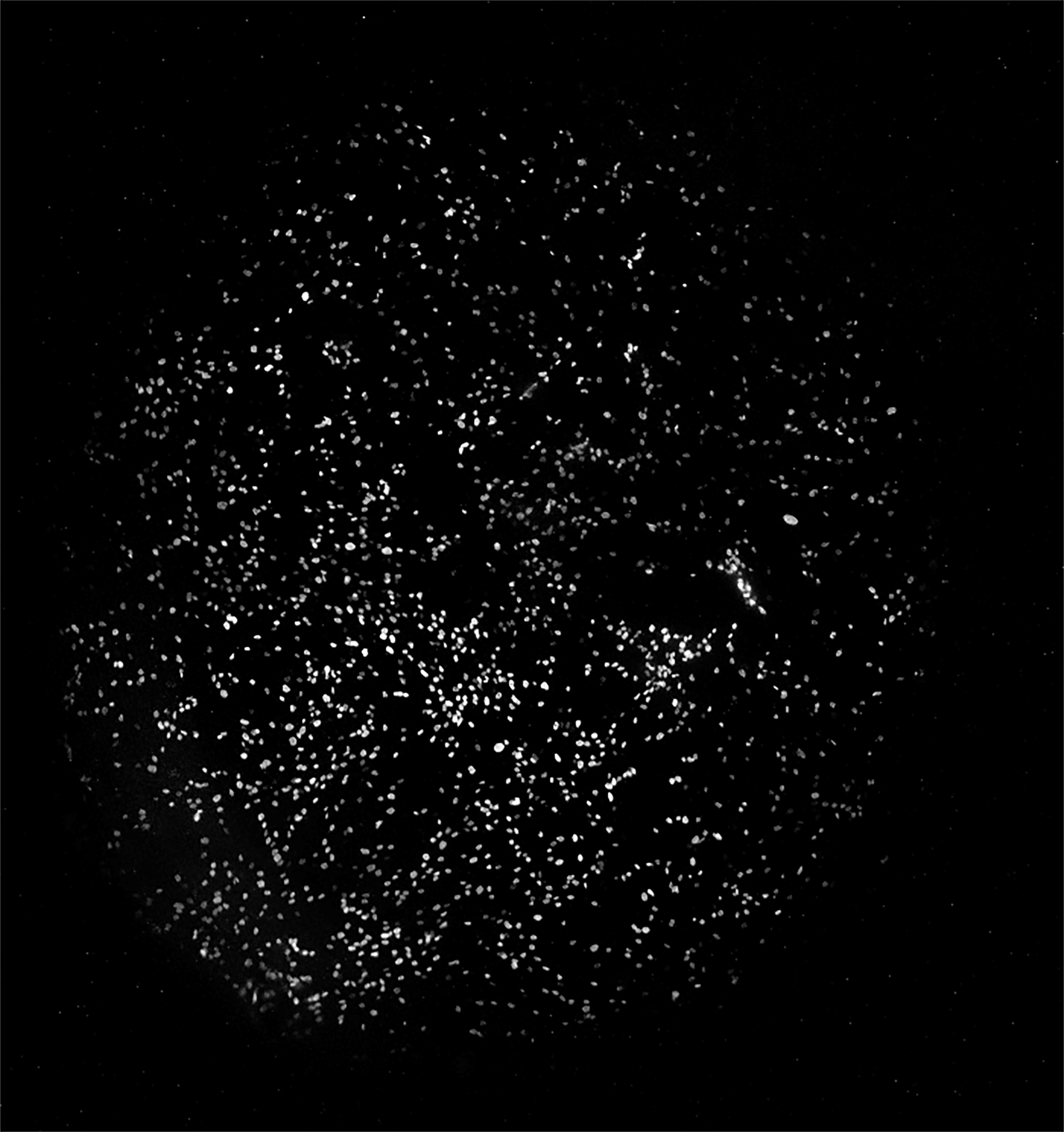

Supplement: Supplementary file 7 — Source data Fig. 5 [file 44321_2024_91_MOESM7_ESM.zip › Figure 5F/27.5V-488.tif]

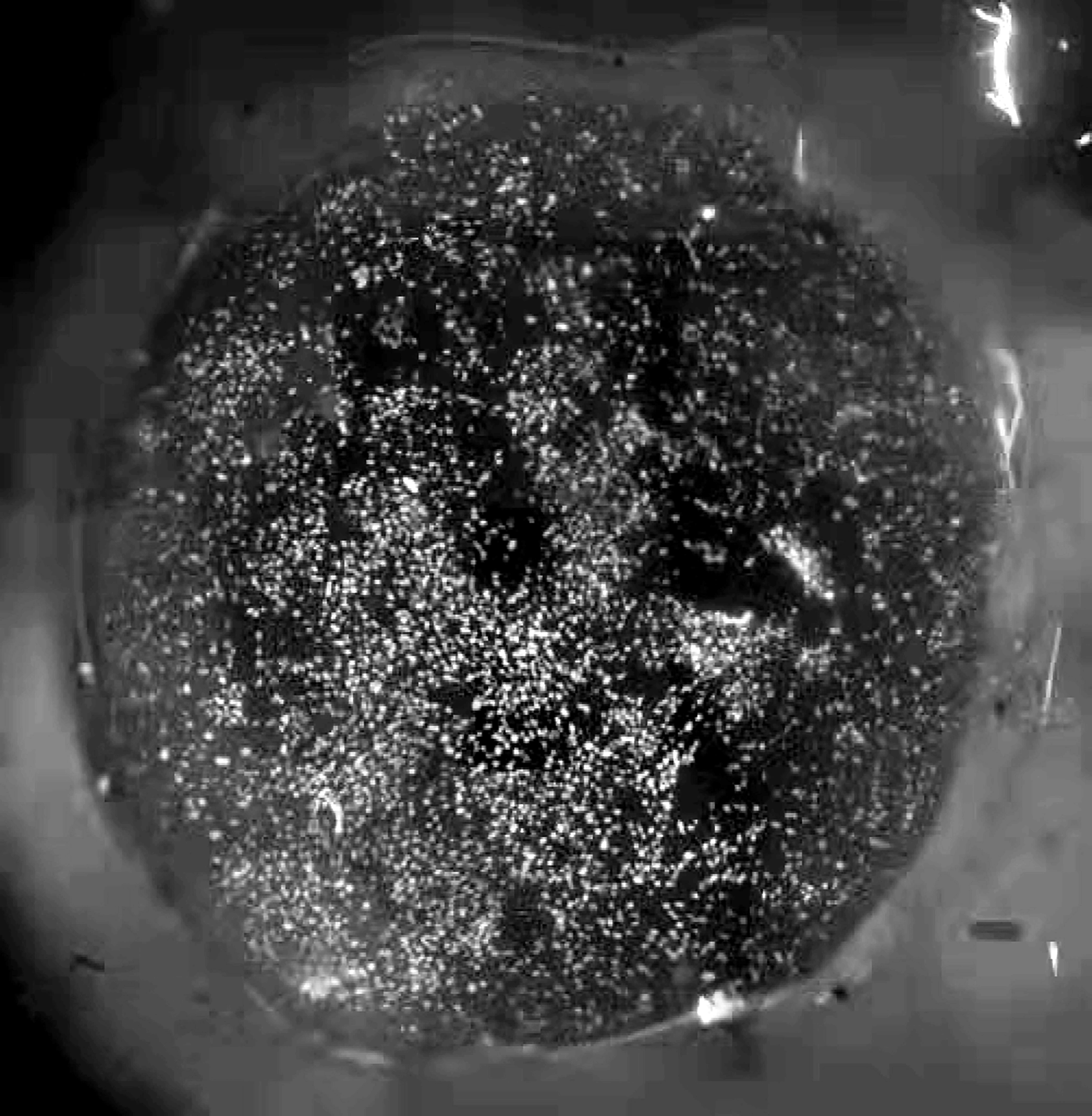

Supplement: Supplementary file 7 — Source data Fig. 5 [file 44321_2024_91_MOESM7_ESM.zip › Figure 5F/27.5V-DAPI.tif]

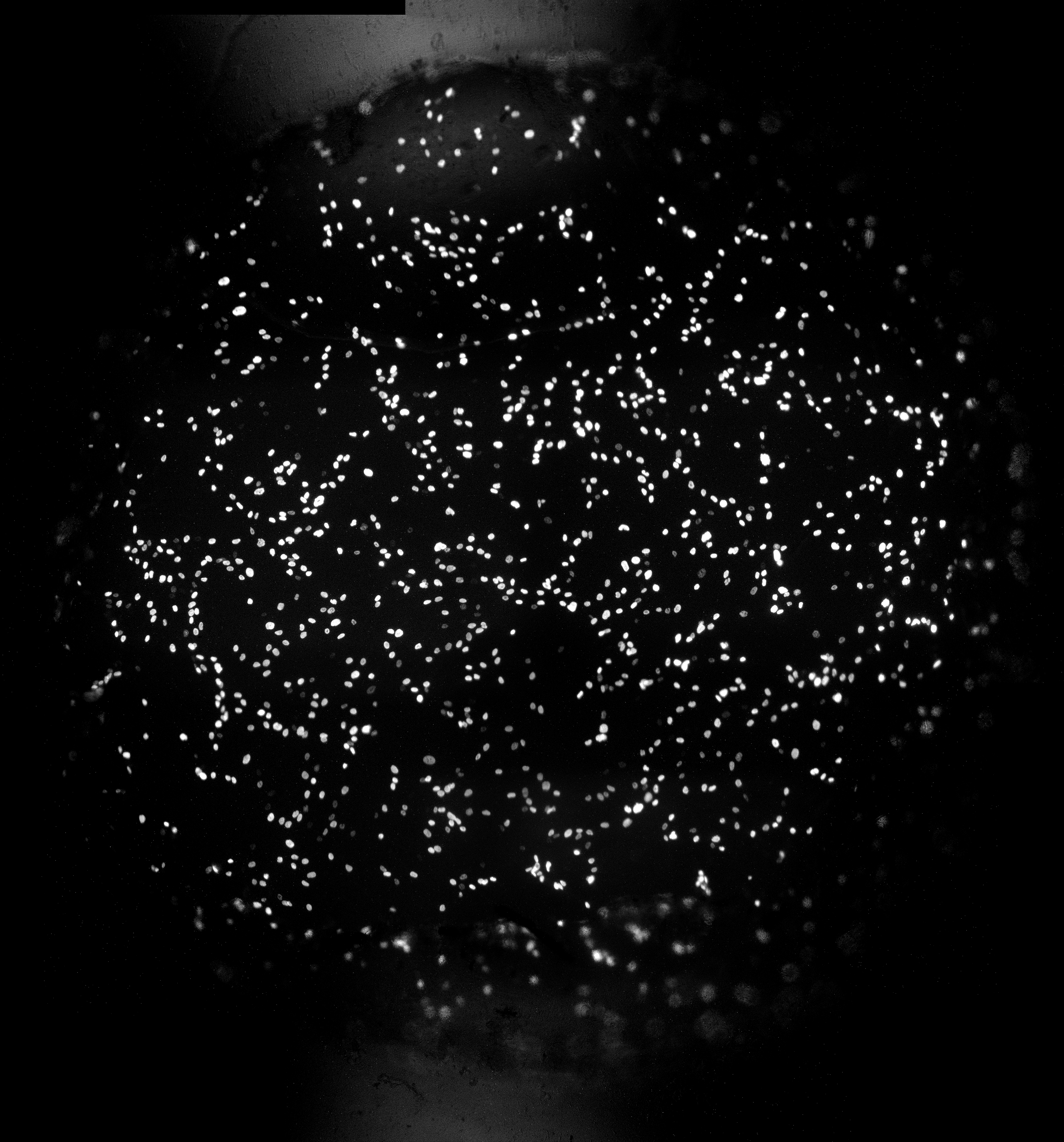

Supplement: Supplementary file 7 — Source data Fig. 5 [file 44321_2024_91_MOESM7_ESM.zip › Figure 5F/42.09V-488.tif]

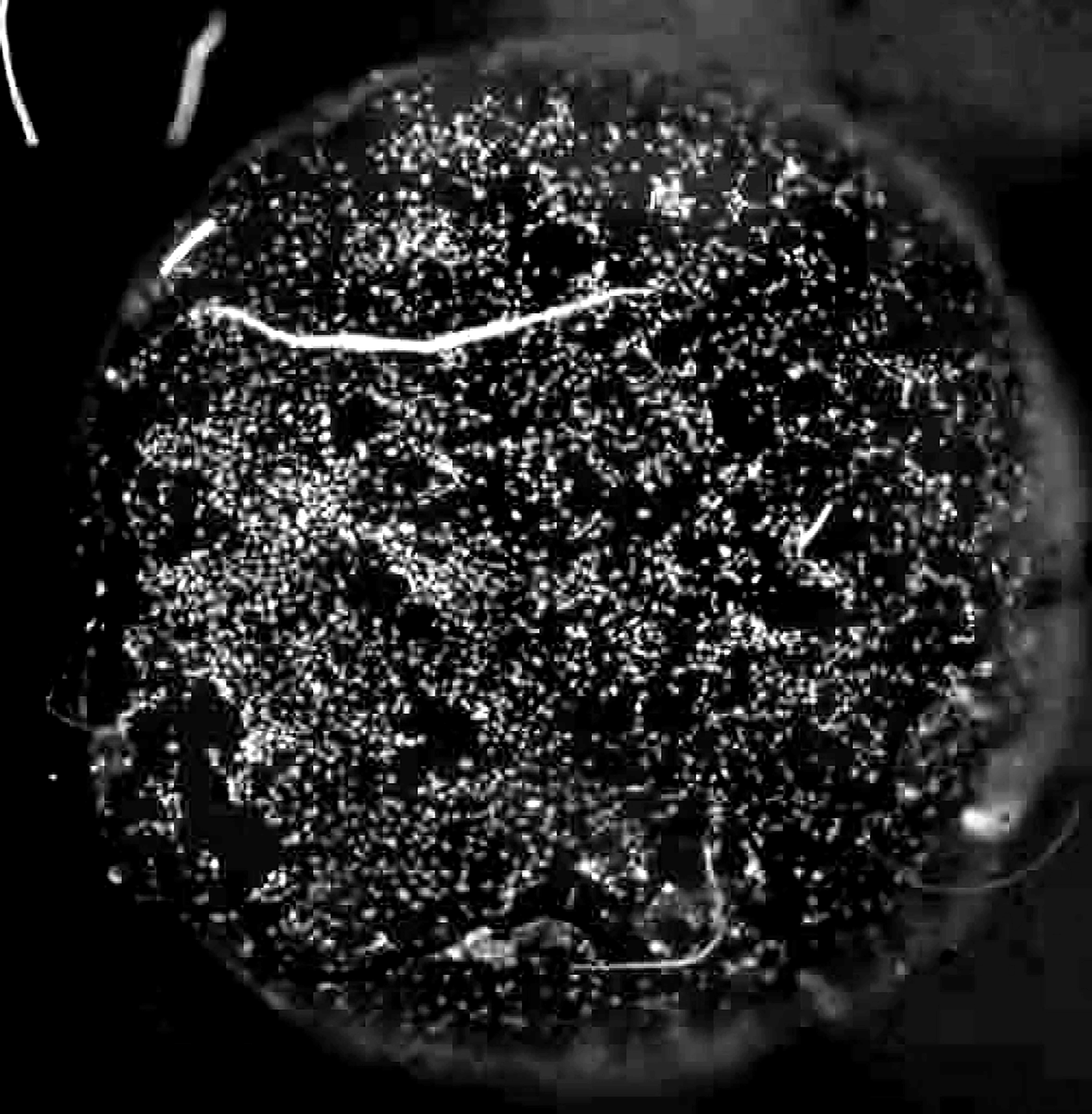

Supplement: Supplementary file 7 — Source data Fig. 5 [file 44321_2024_91_MOESM7_ESM.zip › Figure 5F/42.09V-DAPI.tif]

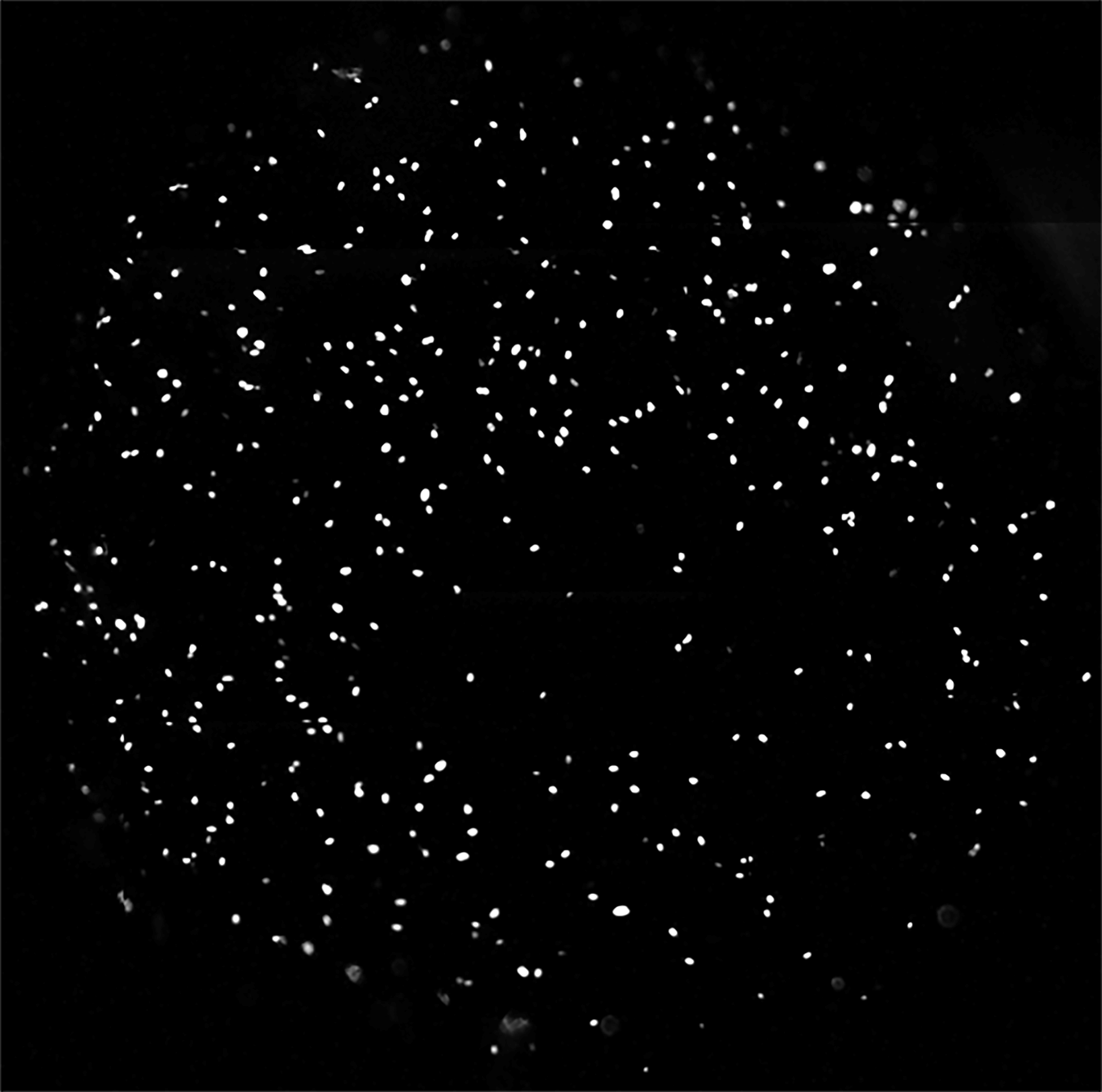

Supplement: Supplementary file 7 — Source data Fig. 5 [file 44321_2024_91_MOESM7_ESM.zip › Figure 5F/558.88V-488.tif]

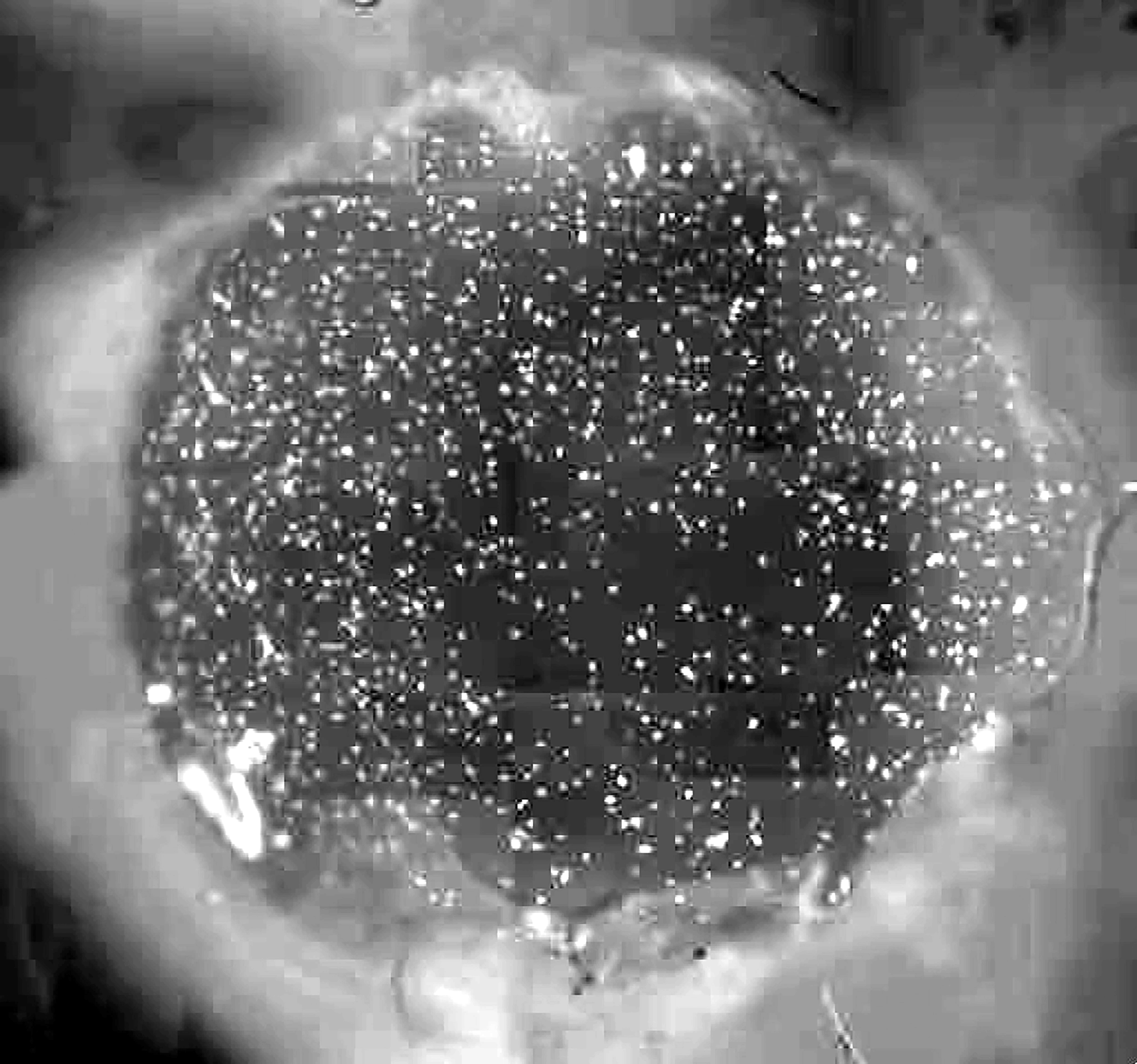

Supplement: Supplementary file 7 — Source data Fig. 5 [file 44321_2024_91_MOESM7_ESM.zip › Figure 5F/558.88V-DAPI.tif]

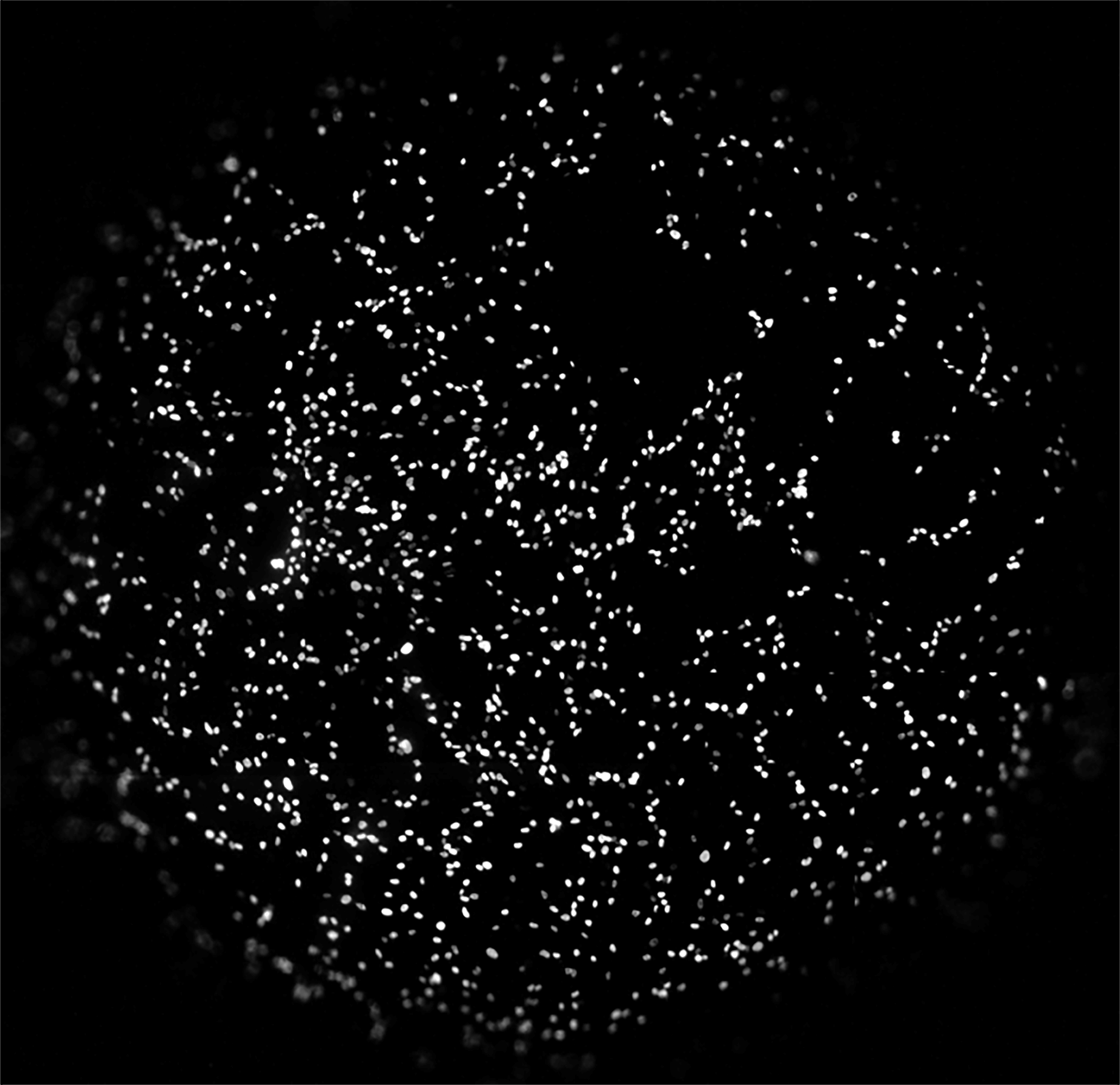

Supplement: Supplementary file 7 — Source data Fig. 5 [file 44321_2024_91_MOESM7_ESM.zip › Figure 5F/75.45V-488.tif]

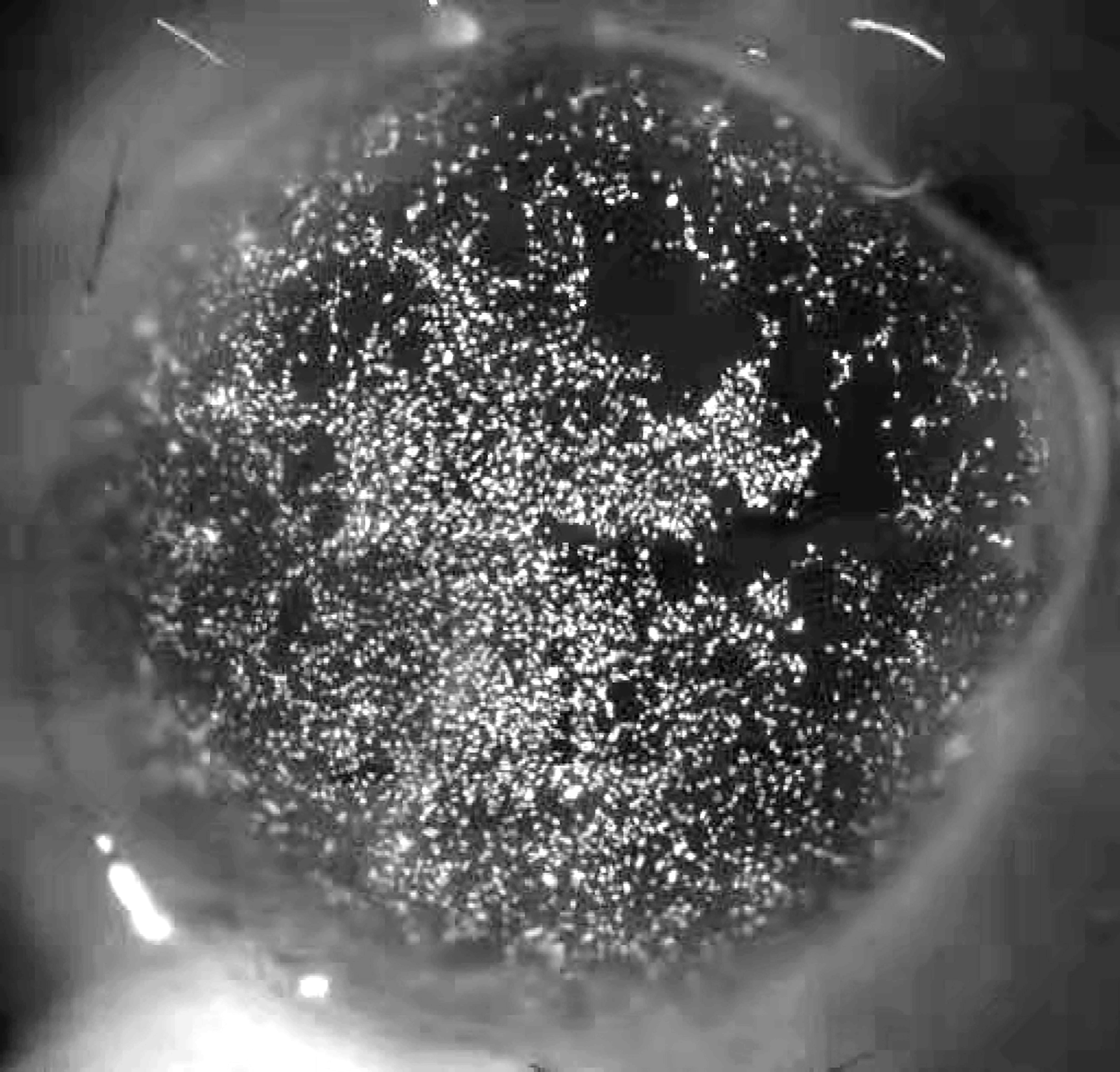

Supplement: Supplementary file 7 — Source data Fig. 5 [file 44321_2024_91_MOESM7_ESM.zip › Figure 5F/75.45V-DAPI.tif]

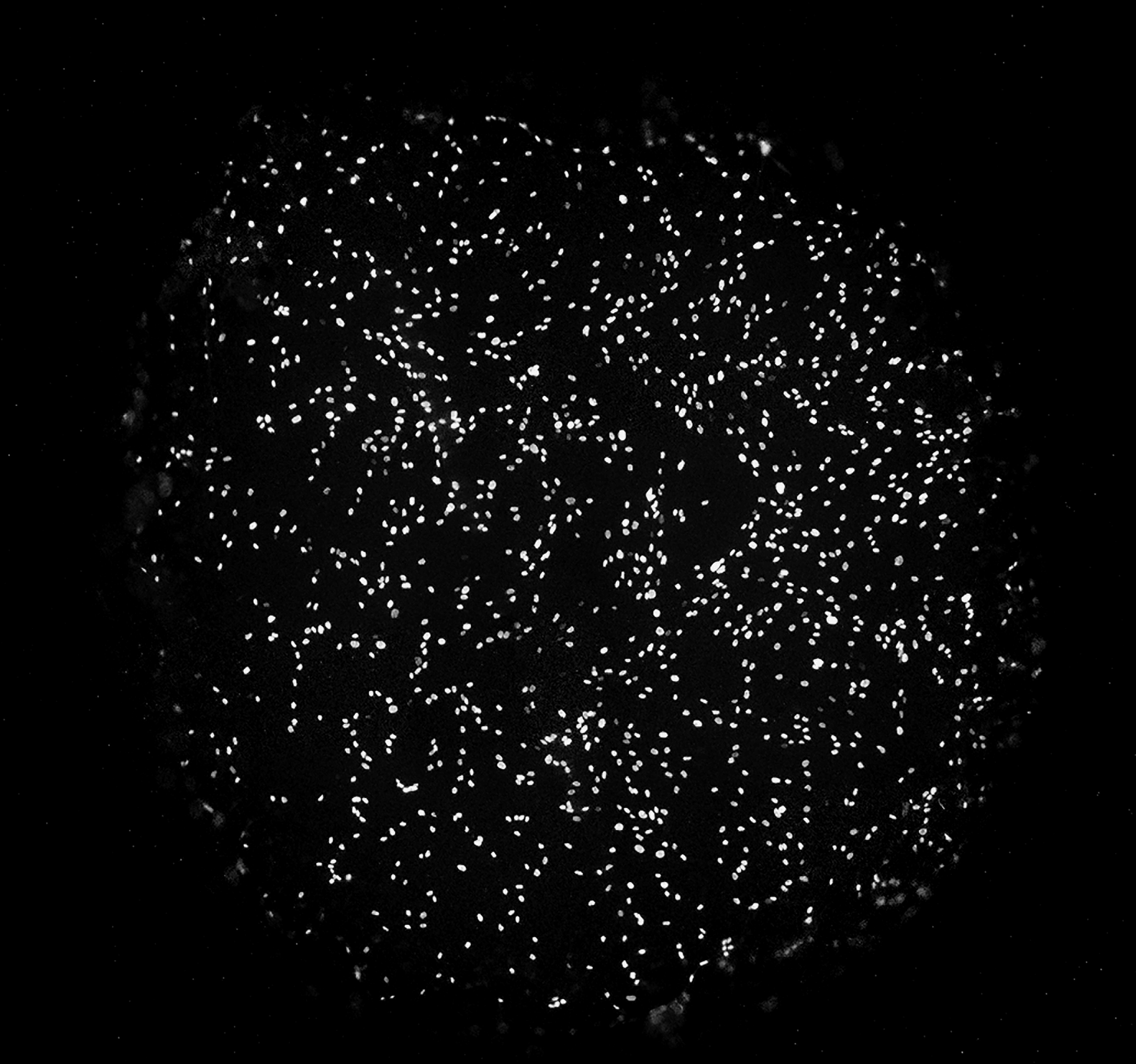

Supplement: Supplementary file 7 — Source data Fig. 5 [file 44321_2024_91_MOESM7_ESM.zip › Figure 5H/0.01¦ÌM-488.tif]

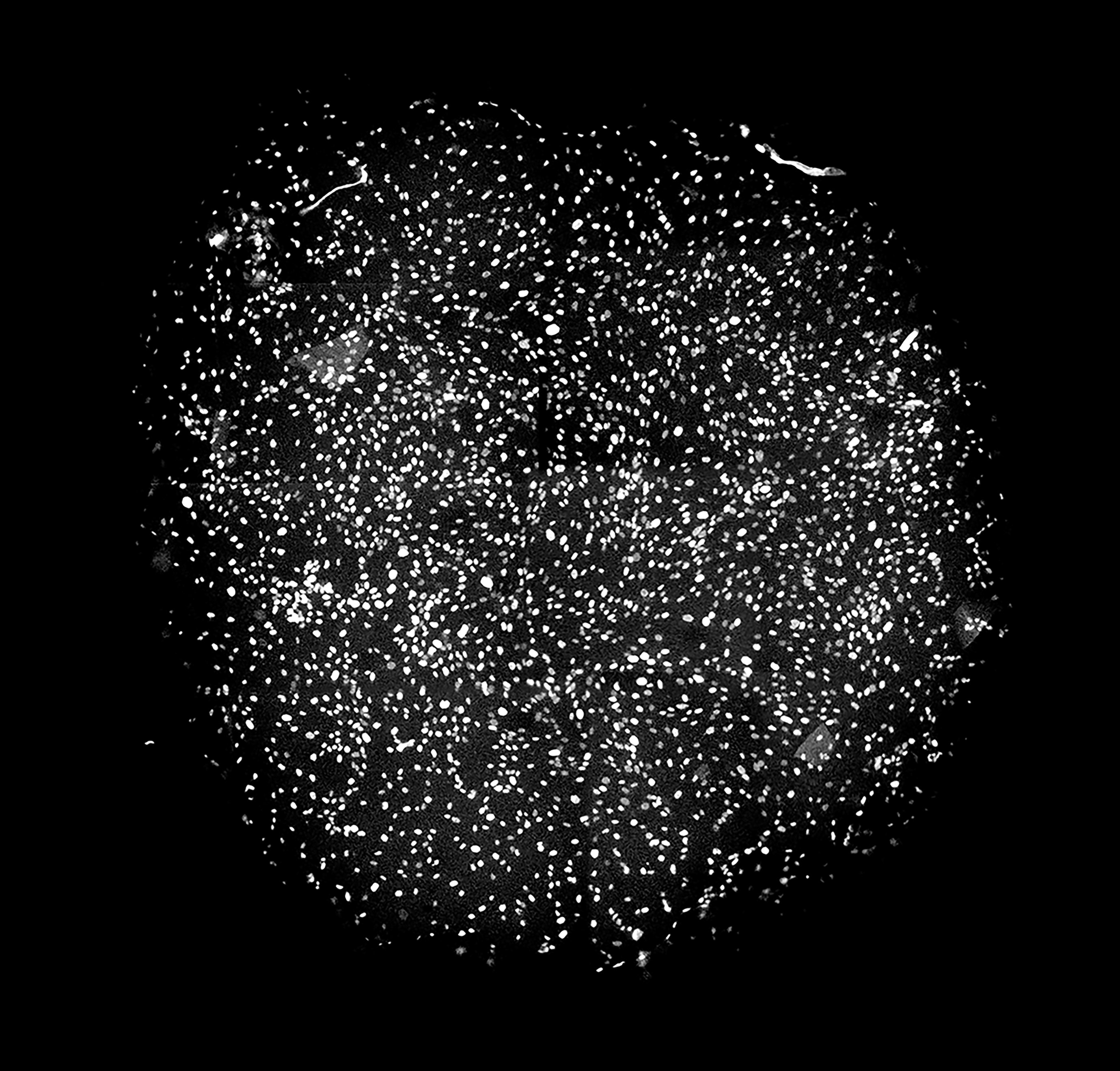

Supplement: Supplementary file 7 — Source data Fig. 5 [file 44321_2024_91_MOESM7_ESM.zip › Figure 5H/0.01¦ÌM-DAPI.tif]

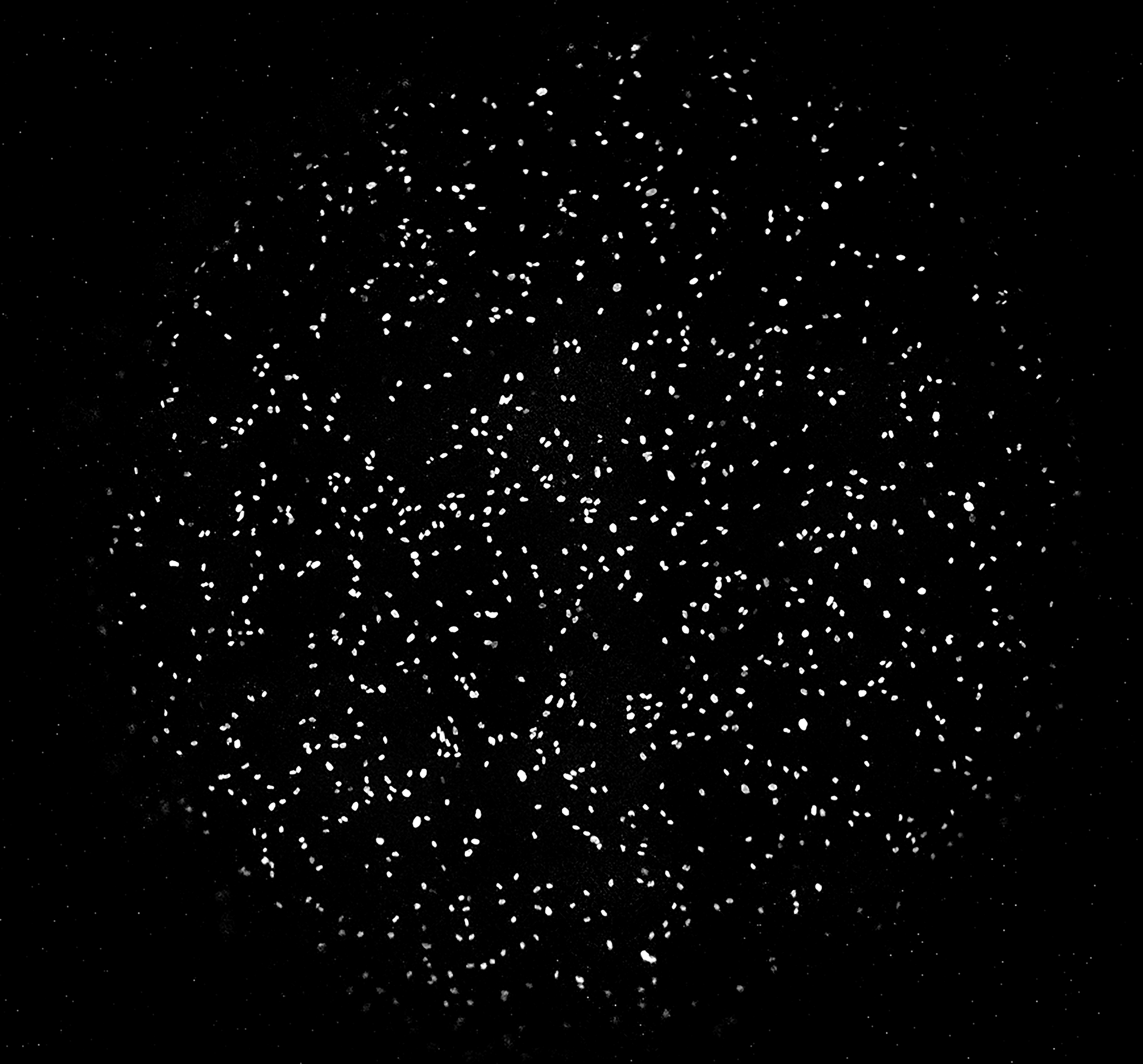

Supplement: Supplementary file 7 — Source data Fig. 5 [file 44321_2024_91_MOESM7_ESM.zip › Figure 5H/0.05¦ÌM-488.tif]

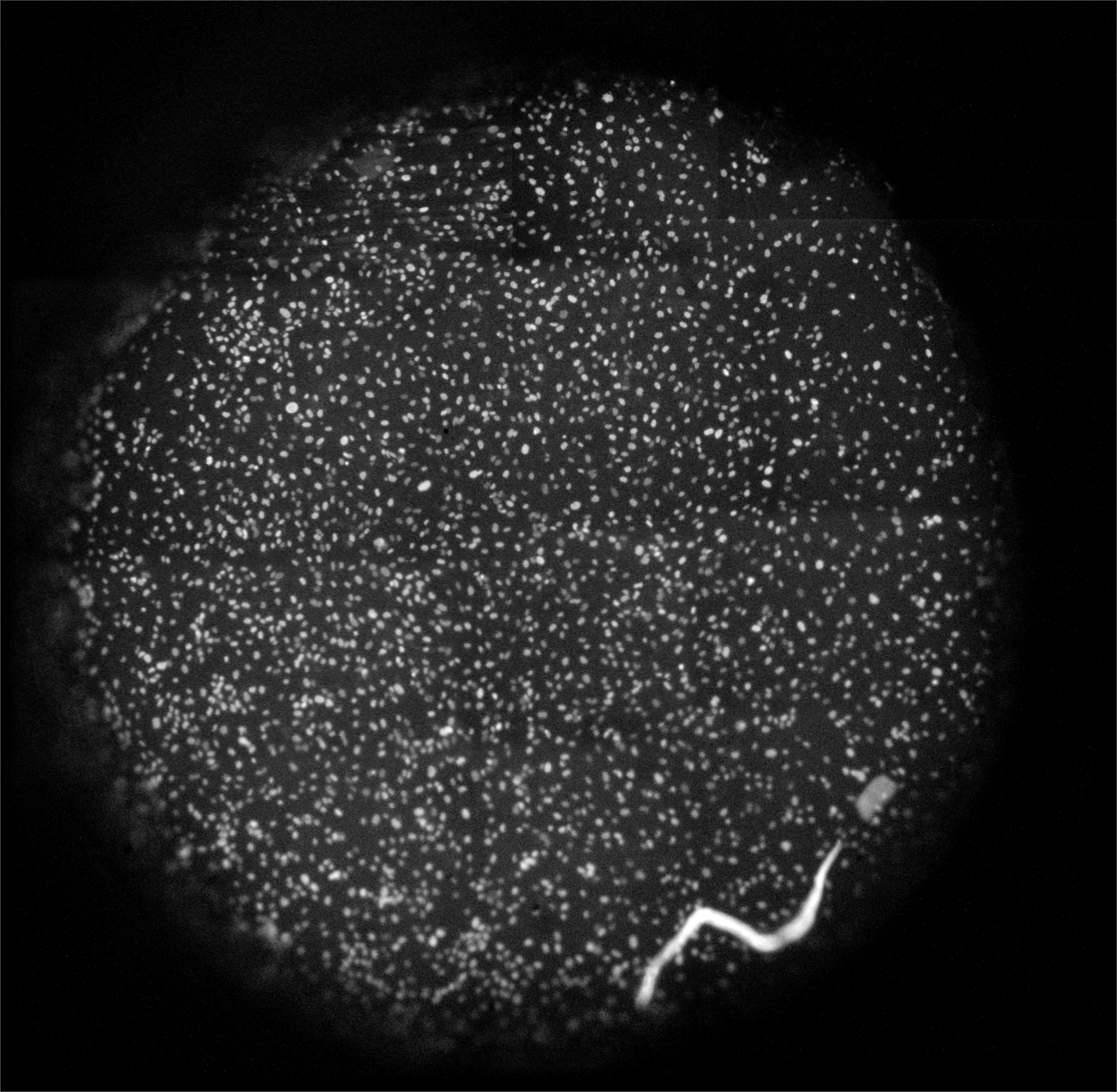

Supplement: Supplementary file 7 — Source data Fig. 5 [file 44321_2024_91_MOESM7_ESM.zip › Figure 5H/0.05¦ÌM-DAPI.tif]

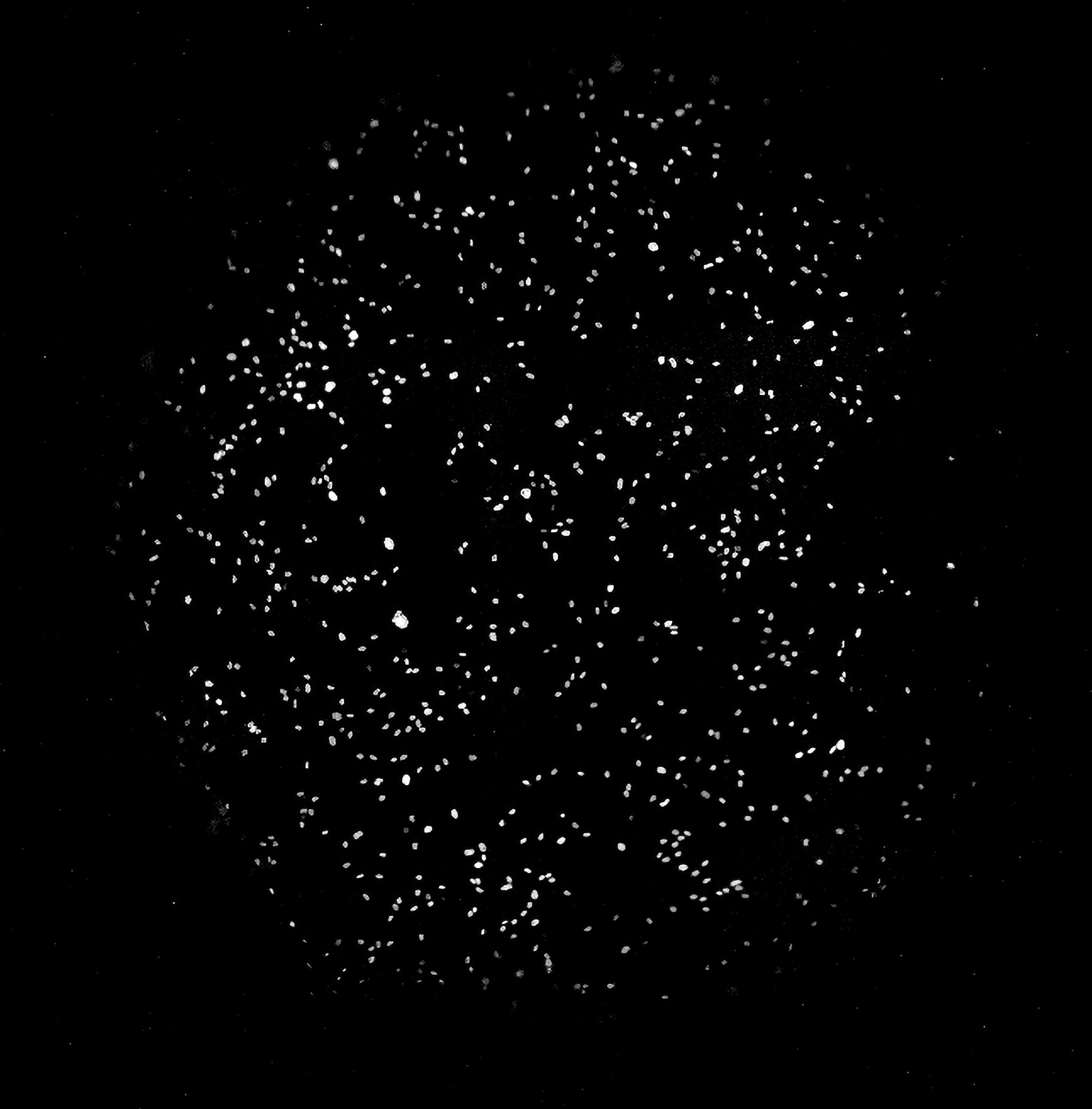

Supplement: Supplementary file 7 — Source data Fig. 5 [file 44321_2024_91_MOESM7_ESM.zip › Figure 5H/0.5¦ÌM-488.tif]

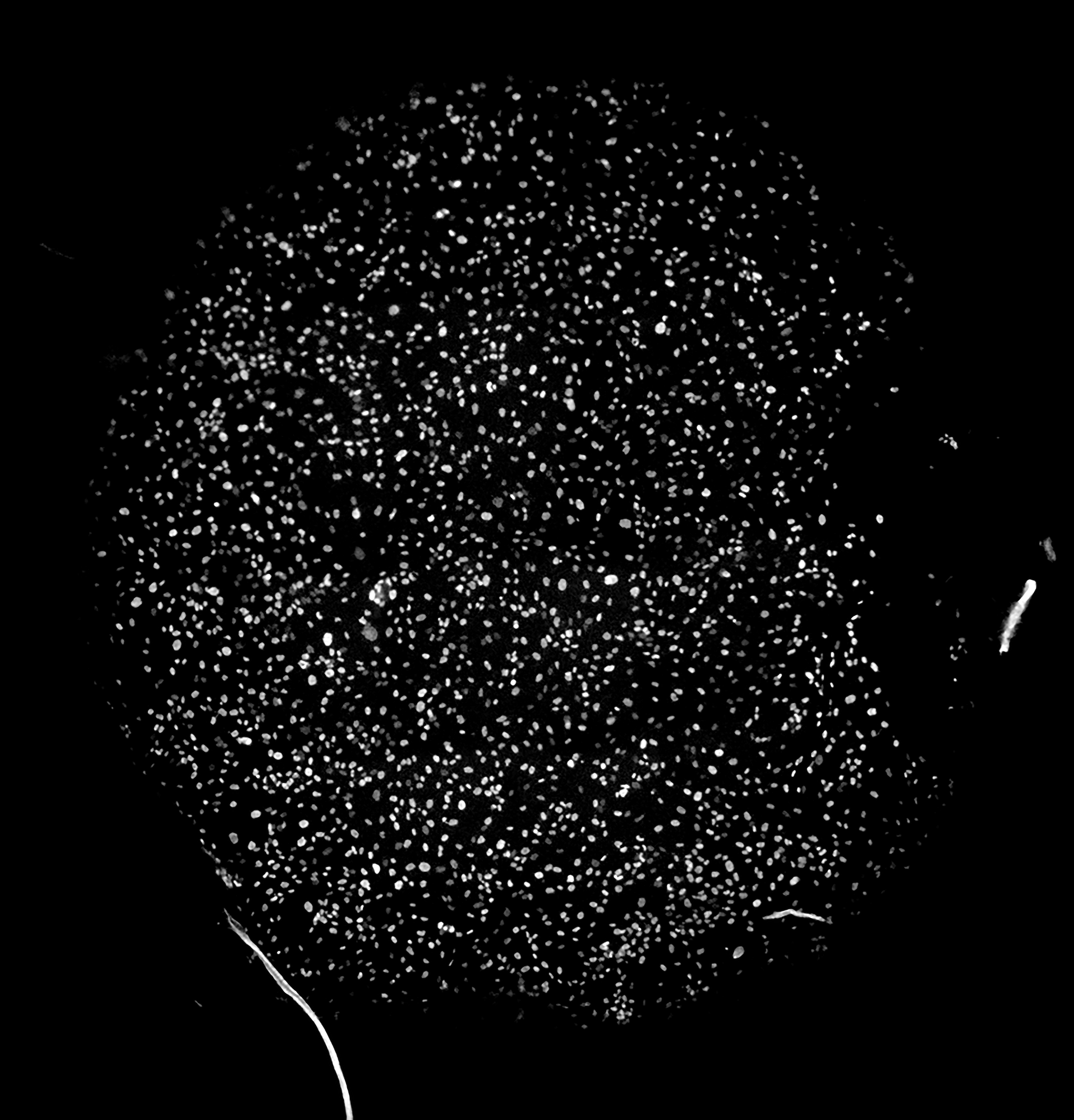

Supplement: Supplementary file 7 — Source data Fig. 5 [file 44321_2024_91_MOESM7_ESM.zip › Figure 5H/0.5¦ÌM-DAPI.tif]

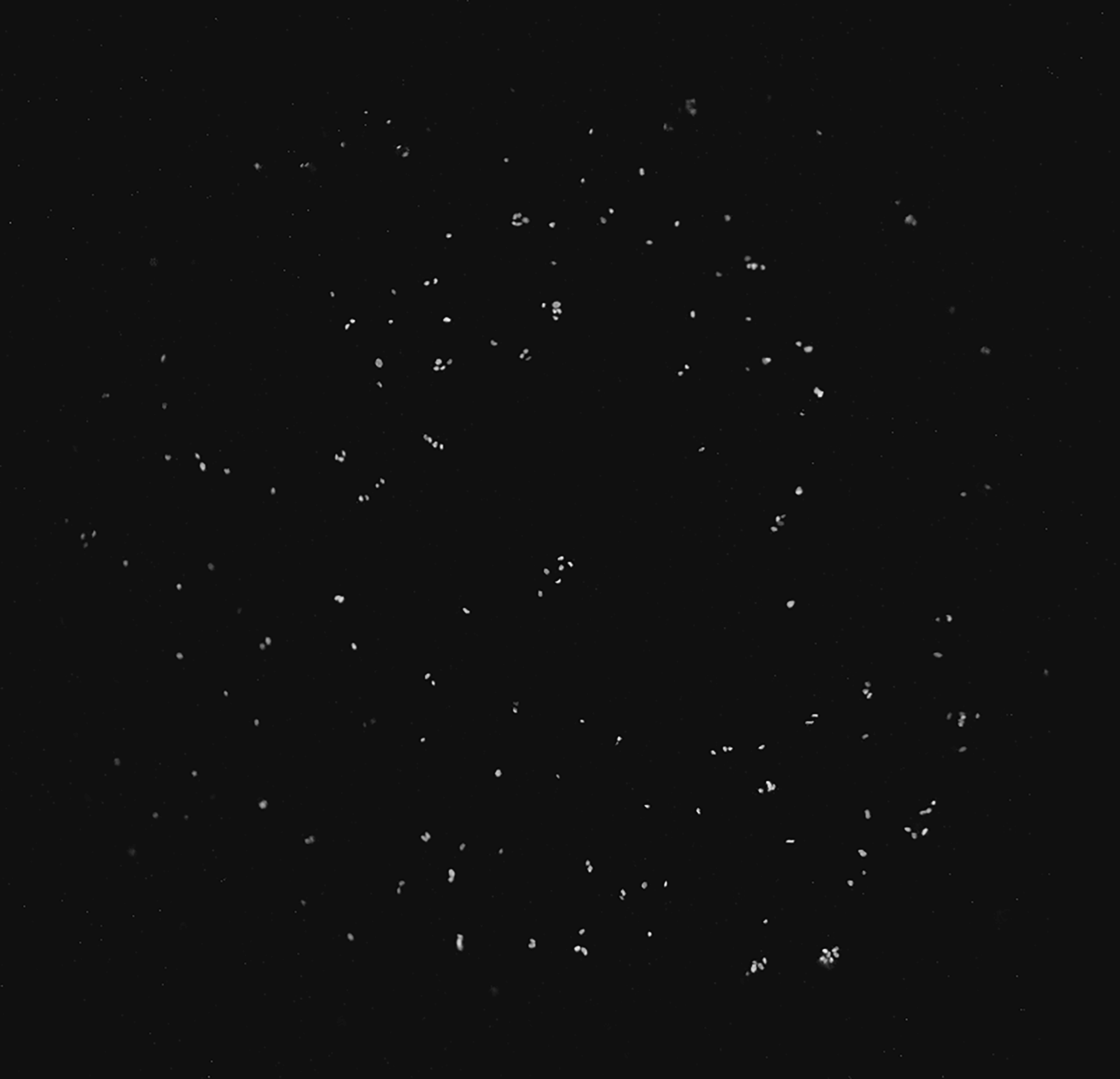

Supplement: Supplementary file 7 — Source data Fig. 5 [file 44321_2024_91_MOESM7_ESM.zip › Figure 5H/15¦ÌM-488.tif]

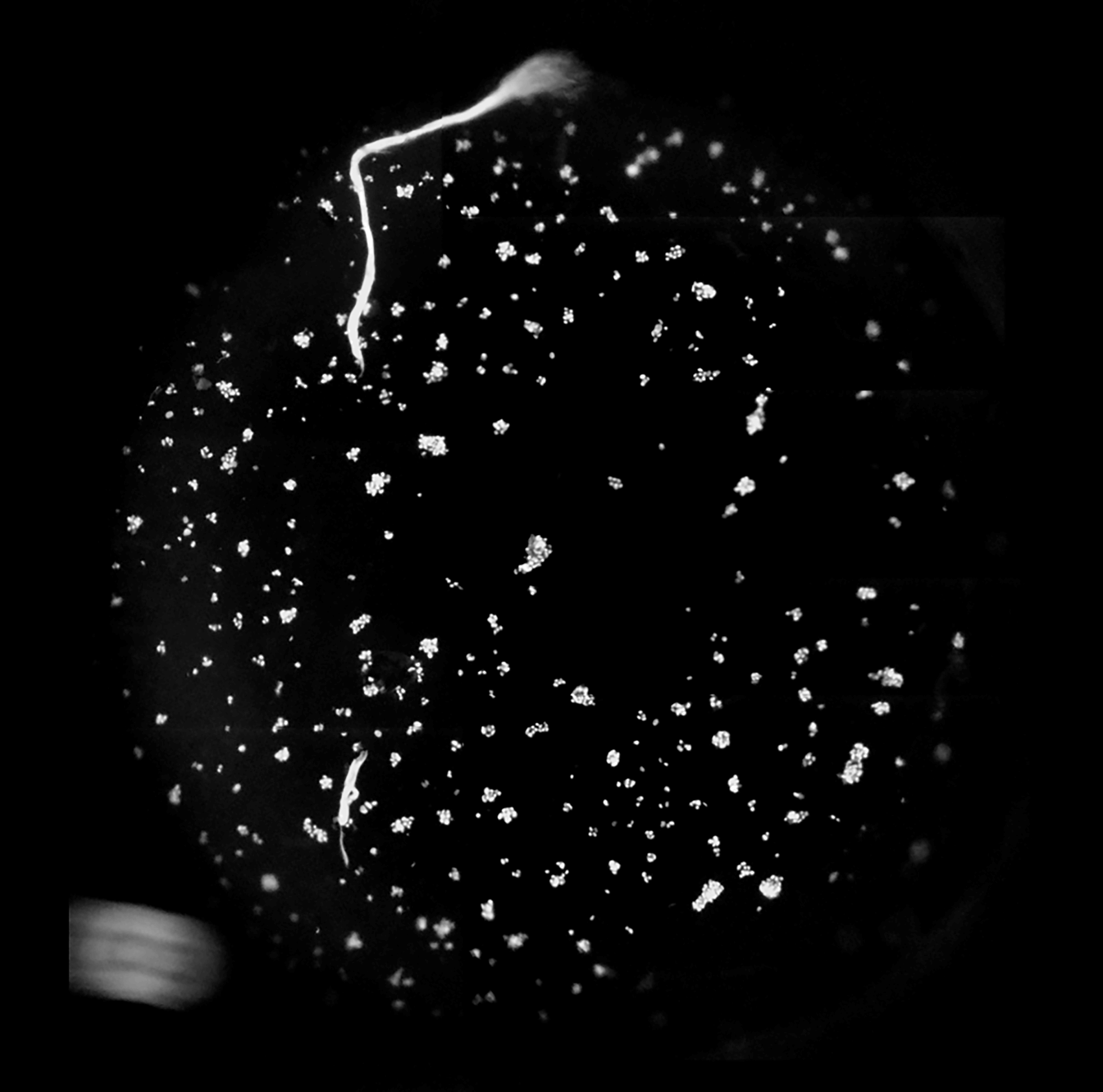

Supplement: Supplementary file 7 — Source data Fig. 5 [file 44321_2024_91_MOESM7_ESM.zip › Figure 5H/15¦ÌM-DAPI.tif]

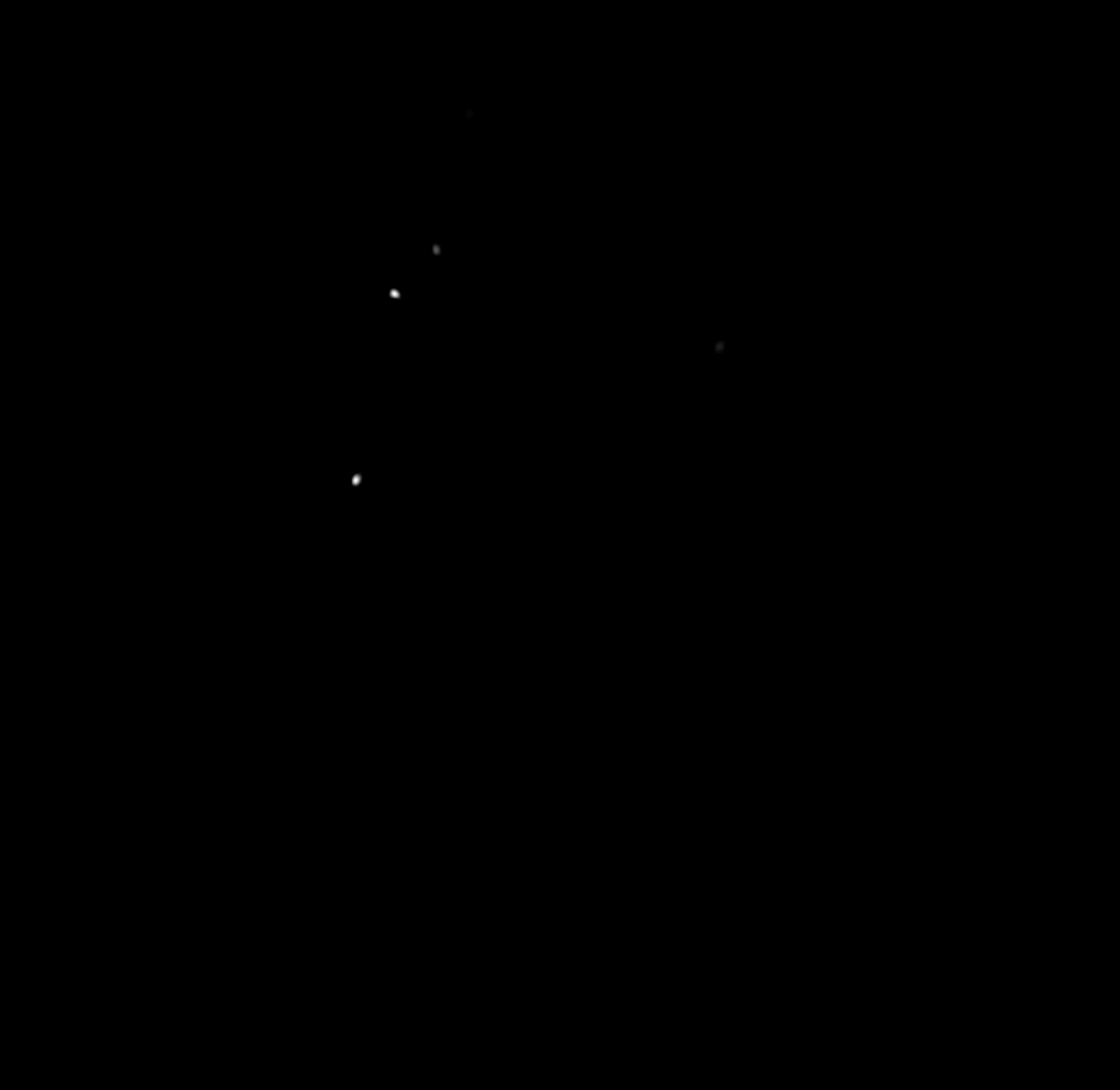

Supplement: Supplementary file 7 — Source data Fig. 5 [file 44321_2024_91_MOESM7_ESM.zip › Figure 5H/30¦ÌM-488.tif]

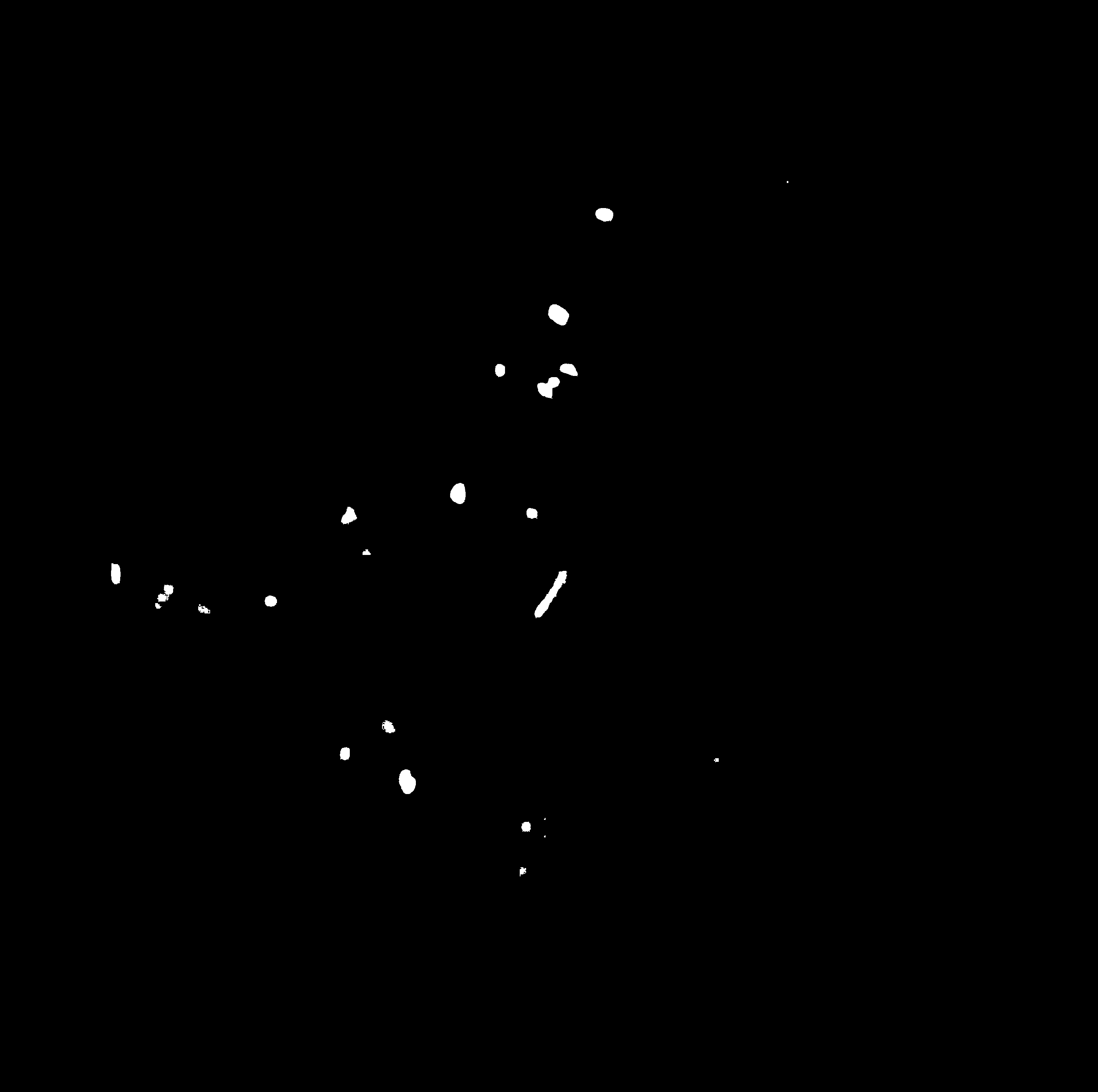

Supplement: Supplementary file 7 — Source data Fig. 5 [file 44321_2024_91_MOESM7_ESM.zip › Figure 5H/30¦ÌM-DAPI.tif]

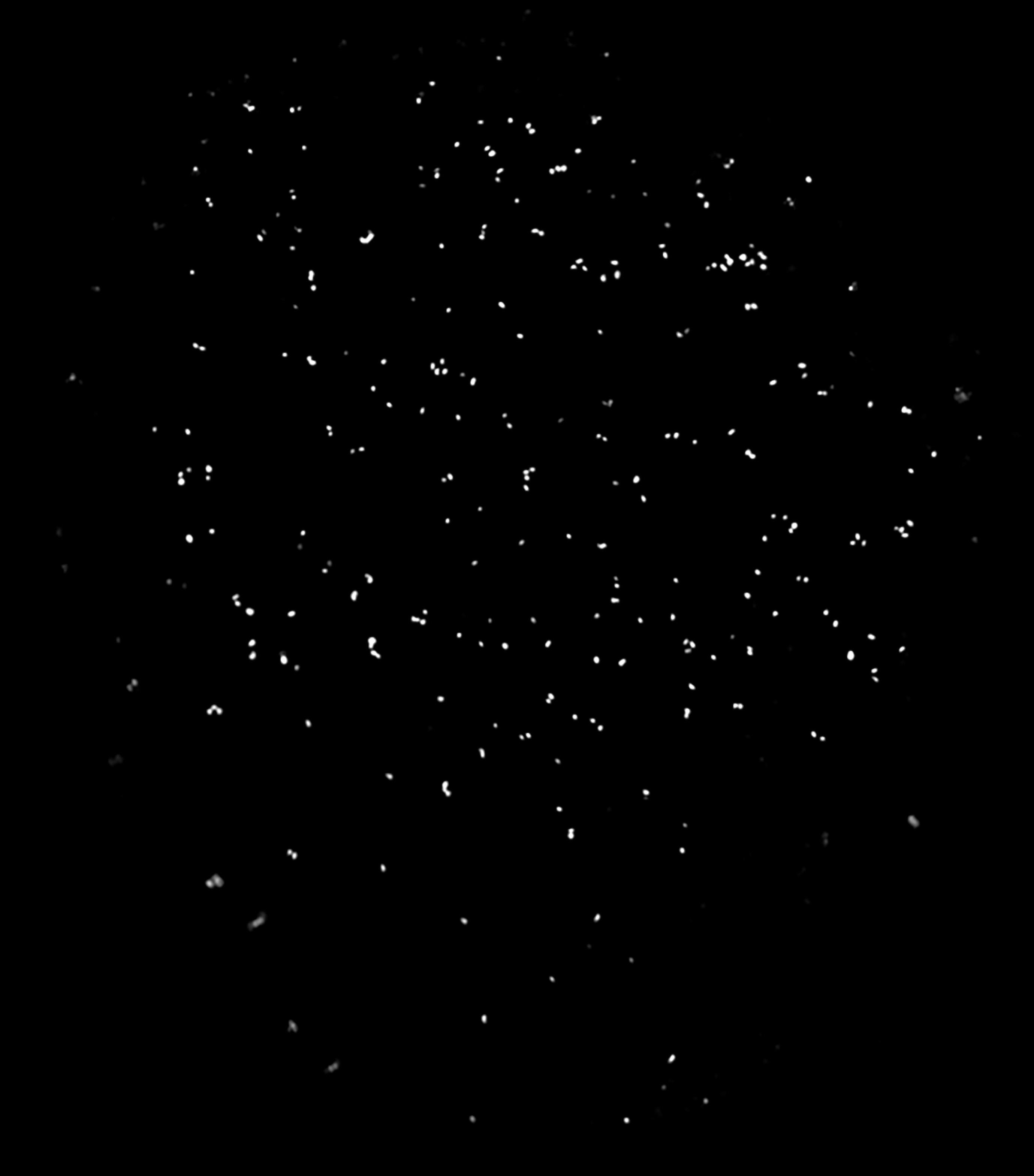

Supplement: Supplementary file 7 — Source data Fig. 5 [file 44321_2024_91_MOESM7_ESM.zip › Figure 5H/5¦ÌM-488.tif]

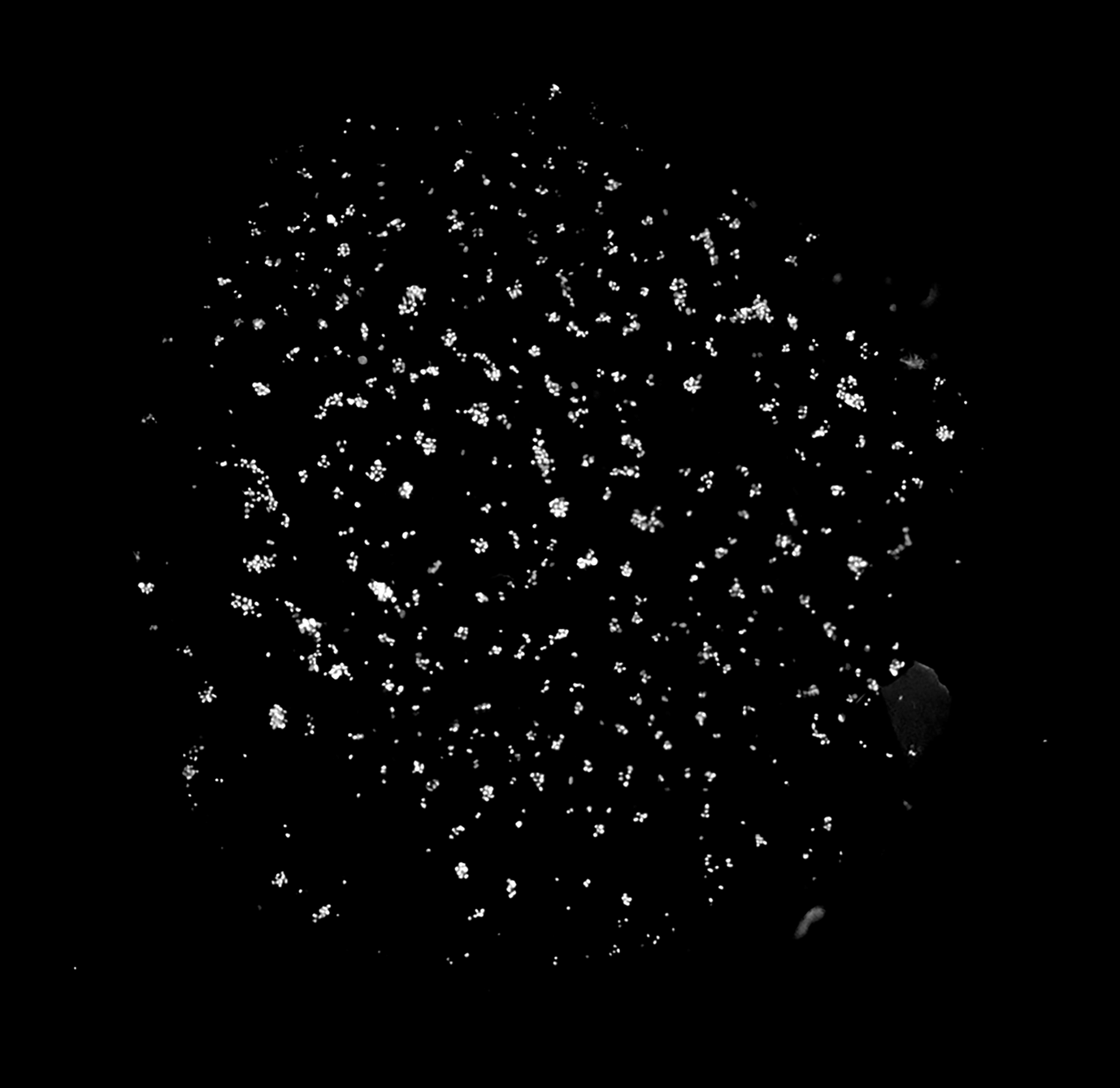

Supplement: Supplementary file 7 — Source data Fig. 5 [file 44321_2024_91_MOESM7_ESM.zip › Figure 5H/5¦ÌM-DAPI.tif]

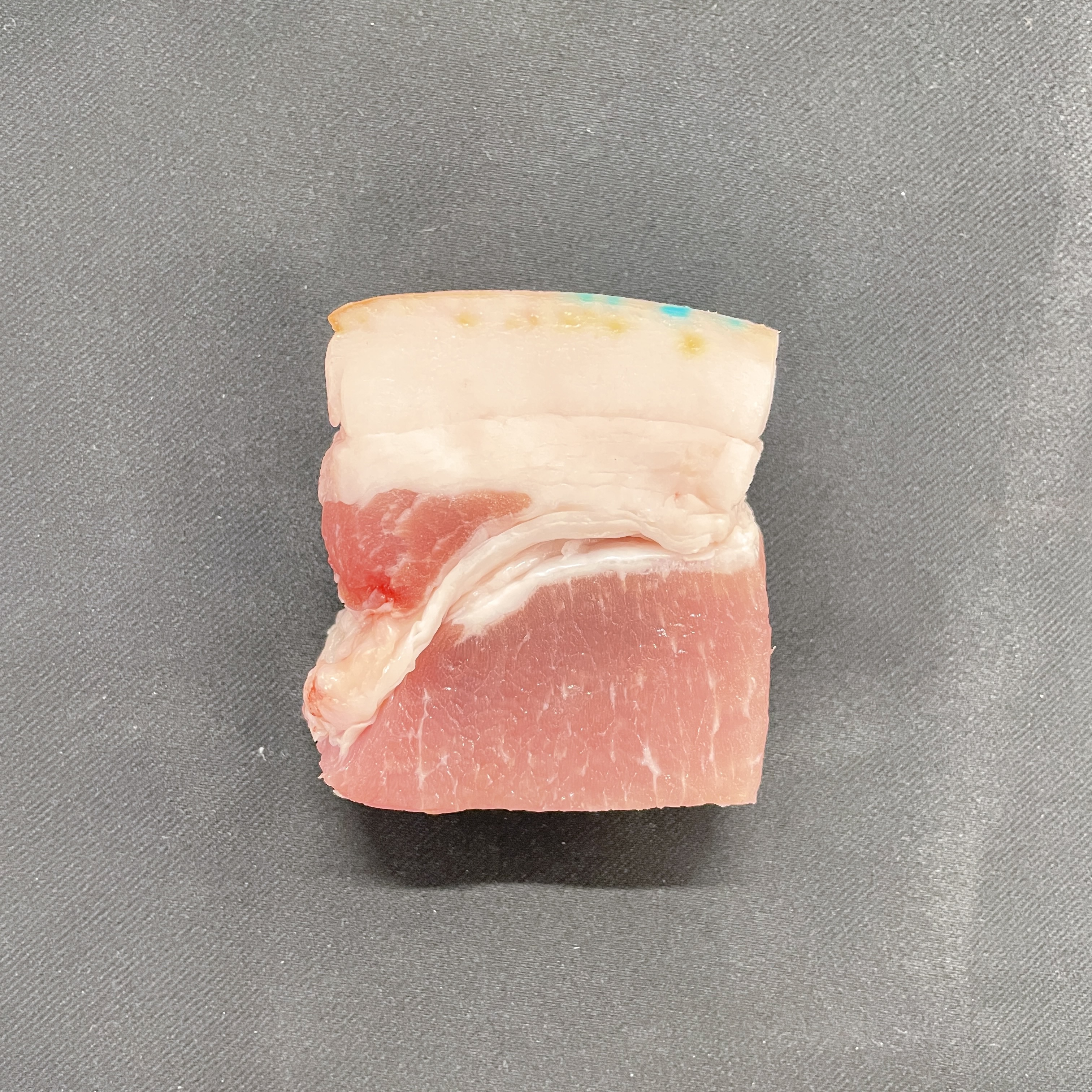

Supplement: Supplementary file 8 — Source data Fig. 6 [file 44321_2024_91_MOESM8_ESM.zip › Figure 6C/Figure 6C.tif]

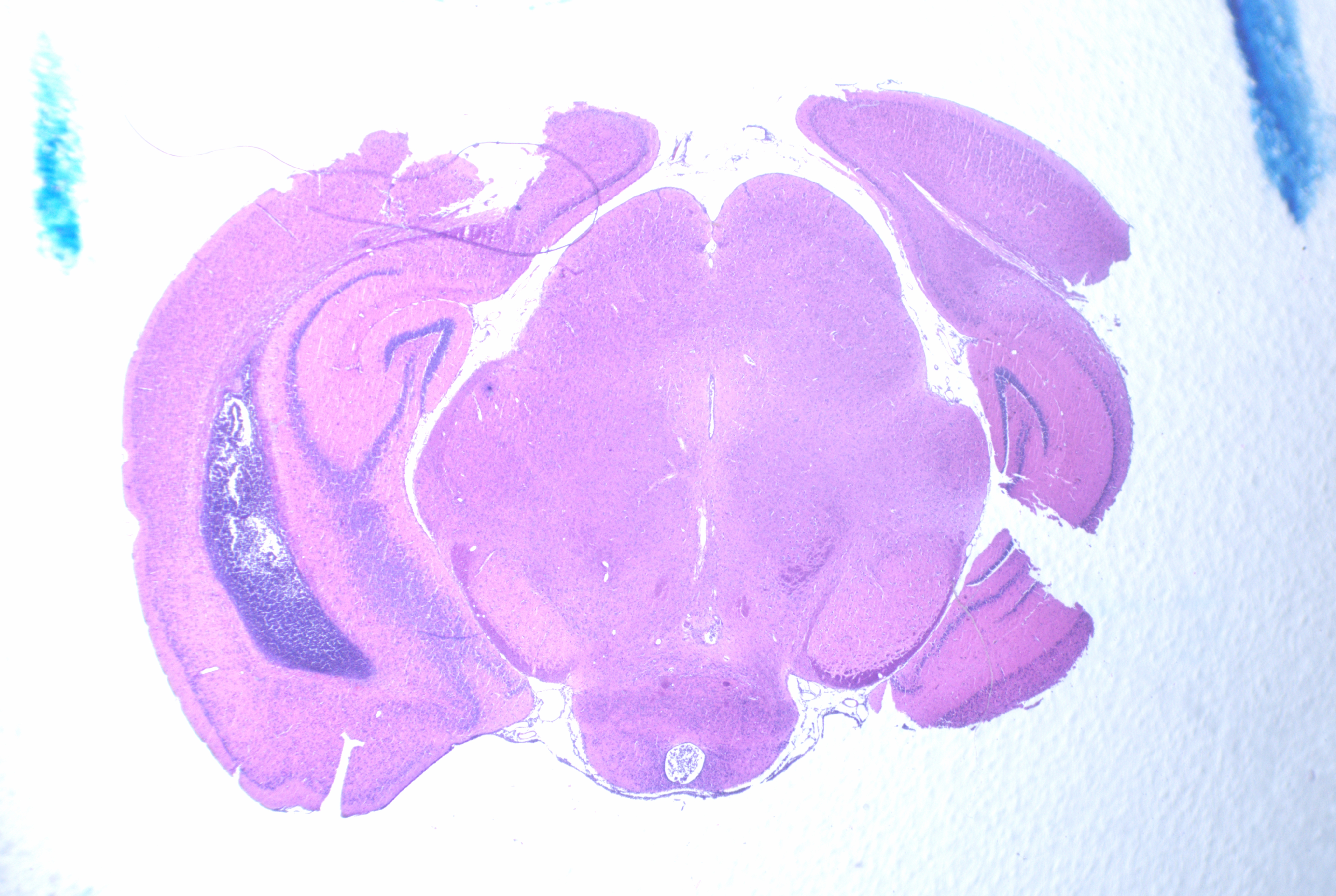

Supplement: Supplementary file 8 — Source data Fig. 6 [file 44321_2024_91_MOESM8_ESM.zip › Figure 6F/Control.tif]

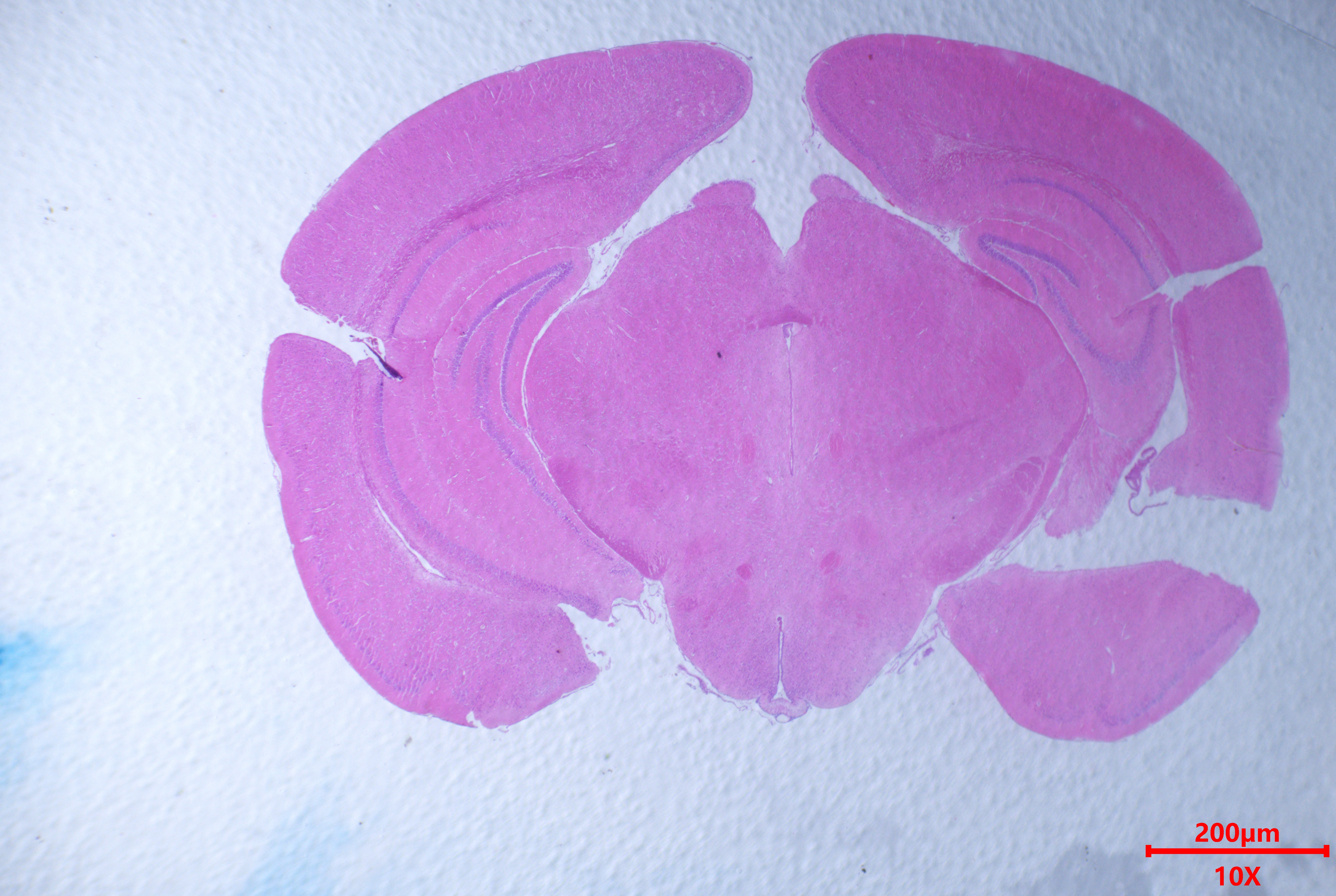

Supplement: Supplementary file 8 — Source data Fig. 6 [file 44321_2024_91_MOESM8_ESM.zip › Figure 6F/MIBTS.tif]
